# Carbon dioxide reforming of methane over Ni-based catalysts: a comprehensive review of coke suppression strategies

**DOI:** 10.1039/d6ra03680e

**Published:** 2026-07-24

**Authors:** Abbas A. Jawad, Sura A. Ahmed, Hasan J. Al-Abedi

**Affiliations:** a Midland Refineries Company MRC, AL Daura Refinery Company, Project Management Division Baghdad Iraq abbasajd5d@outlook.com abbasajd5d@gmail.com; b Department of Chemical and Biochemical Engineering, Missouri University of Science and Technology Rolla MO 65409-1230 USA; c Midland Refineries Company MRC, AL Daura Refinery Company, Maintenance Board Baghdad Iraq

## Abstract

Catalytic dry reforming of methane (DRM) can provide aids for turning two important greenhouse gases methane and carbon dioxide (CH_4_ and CO_2_) into valuable synthesis gas. For Ni-based catalysts – the most promising because of low costs and high activity industrial applications are severely limited by fast deactivation due to carbon deposition (coking). A focused and critical assessment of coke suppression strategies for Ni based DRM catalysts is provided in this review. Our systematic review includes: (1) basic (MgO) and redox-active (CeO_2_ ZrO_2_) support roles in CO_2_ activation and carbon gasification; (2) noble (Pt, Ru) and non-noble (Fe, Co, Mo) promoters altering the surface chemistry of Ni; (3) bi- and trimetallic formulations providing synergistic anti-coking interfaces; (4) thermodynamics and kinetics of coke formation; (5) catalyst deactivation mechanisms; regeneration methods; (6) the effect of synthesis techniques (impregnation,-and co precipitation sol–gel) on Ni dispersion on coke resistance. We critically interrogate and compare key contradictory findings within the literature-including its dominant source of coke (methane cracking *vs.* Boudouard reaction), and most beneficial promoter (Pt *vs.* Ru). Data from the literature suggest a range of reported coke deposition rates (15 mg C g_cat^−1^ h^−1^) as well as catalyst stability durations (20–200 h under 1 bar conditions), each in significant deficit of industrial targets (>8000 h on stream, 10 bar), resistance to trace impurities (H_2_S), and pilot scale tests, outlining a route to durable, coke resistant Ni-based catalysts for sustainable DRM.

## Abstract 2

Bifunctional reaction mechanism of DRM over Pt/Mo–Fe/Ni/Al_2_O_3_–CeO_2_.

## Graphical abstract

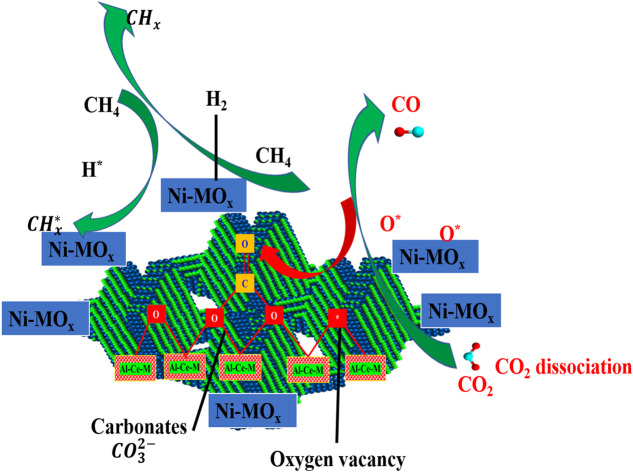

## 1. Introduction

### 1.1. Challenges and opportunities

Therefore, this review aims to provide a comprehensive and critical examination of the current state of nickel-based catalysts doped with metal oxides for the dry reforming of methane. This review provides a comprehensive and critical assessment of the state-of-the-art in designing coke-resistant Ni-based catalysts for DRM. The central objective is to analyze the fundamental principles and recent breakthroughs in suppressing carbon formation. We systematically evaluate three primary levers of catalyst design:

1. The catalyst support: examining how basic oxides (*e.g.*, MgO) and redox-active oxides (*e.g.*, CeO_2_, ZrO_2_) participate in CO_2_ activation and carbon gasification through strong metal–support interactions (SMSI).

2. Promoters: analyzing how the addition of noble and non-noble metals alters the electronic and geometric properties of Ni to inhibit C–H bond cleavage or enhance C–O bond formation.

3. Multi-metallic formulations: discussing the synergistic effects in bi- and trimetallic catalysts that lead to superior coke resistance compared to monometallic Ni. Furthermore, we dissect the mechanisms of catalyst deactivation, with a detailed thermodynamic and kinetic analysis of coke formation pathways. Finally, we survey strategies for catalyst regeneration and the sustainable reclamation of valuable metals from spent catalysts. By identifying key knowledge gaps and outlining future research directions such as the need for *in situ* characterization and atomic-scale catalyst design this review aims to provide a roadmap for developing robust, durable, and economically viable Ni-based catalysts, ultimately unlocking the industrial potential of the DRM process for a sustainable carbon economy.

## 2. Carbon dioxide as a building block for chemical structure

### 2.1. Reaction thermodynamics

In recent years, dry reforming of methane (DRM) has garnered significant attention, driven by both its potential for synthesis gas production and its environmental benefits. Unlike methane steam reforming (MSR), which yields a higher syngas (H_2_/CO) ratio, DRM produces synthesis gas with a lower H_2_/CO ratio. This makes DRM-derived syngas particularly suitable for olefin hydroformylation and carbonylation reactions. Furthermore, DRM shows considerable promise for energy transformation and storage applications due to its reversibility through methanation.^1^ Thermodynamic analysis of DRM reactions provides a foundation for both experimental and computational studies aimed at enhancing product yield. Moreover, it is crucial for understanding the operational boundaries and constraints of the process. Understanding the thermodynamics of dry reforming of methane is essential for optimizing the reaction conditions and improving overall efficiency, as detailed below:^2^

Eqn (1) illustrates an effective method for the valorization of methane (CH_4_) and CO_2_.

1 CH_4_ + CO_2_ → 2CO + 2H_2_ Δ*H*_298_ = 247 kJ mol^−1^

Regarding the application of DRM for syngas production, its industrial implementation has been hindered primarily by the following issues: (i) the concurrent occurrence of the reverse water gas shift reaction (RWGS), as shown in eqn (2), which consumes hydrogen (H_2_) and consequently reduces the H_2_/CO ratio.

2 CO_2_ + H_2_ → CO + H_2_O Δ*H*_298_ = 41 kJ mol^−1^

(ii) Catalyst deactivation and/or reactor plugging caused by carbon deposition resulting from the Boudouard reaction eqn (3), methane (CH_4_) dissociation eqn (4), and the hydrogenation of CO_2_ and carbon monoxide (CO) eqn (5) and (6).

3 2CO → CO_2_ + C Δ*H*_298_ = −172 kJ mol^−1^

4 CH_4_ → C + 2H_2_ Δ*H*_298_ = 75 kJ mol^−1^

5 CO_2_ + 2H_2_ → C + H_2_O Δ*H*_298_ = −90 kJ mol^−1^

6 CO + H_2_ → C + H_2_O Δ*H*_298_ = −131.3 kJ mol^−1^

**Fig. 1:**
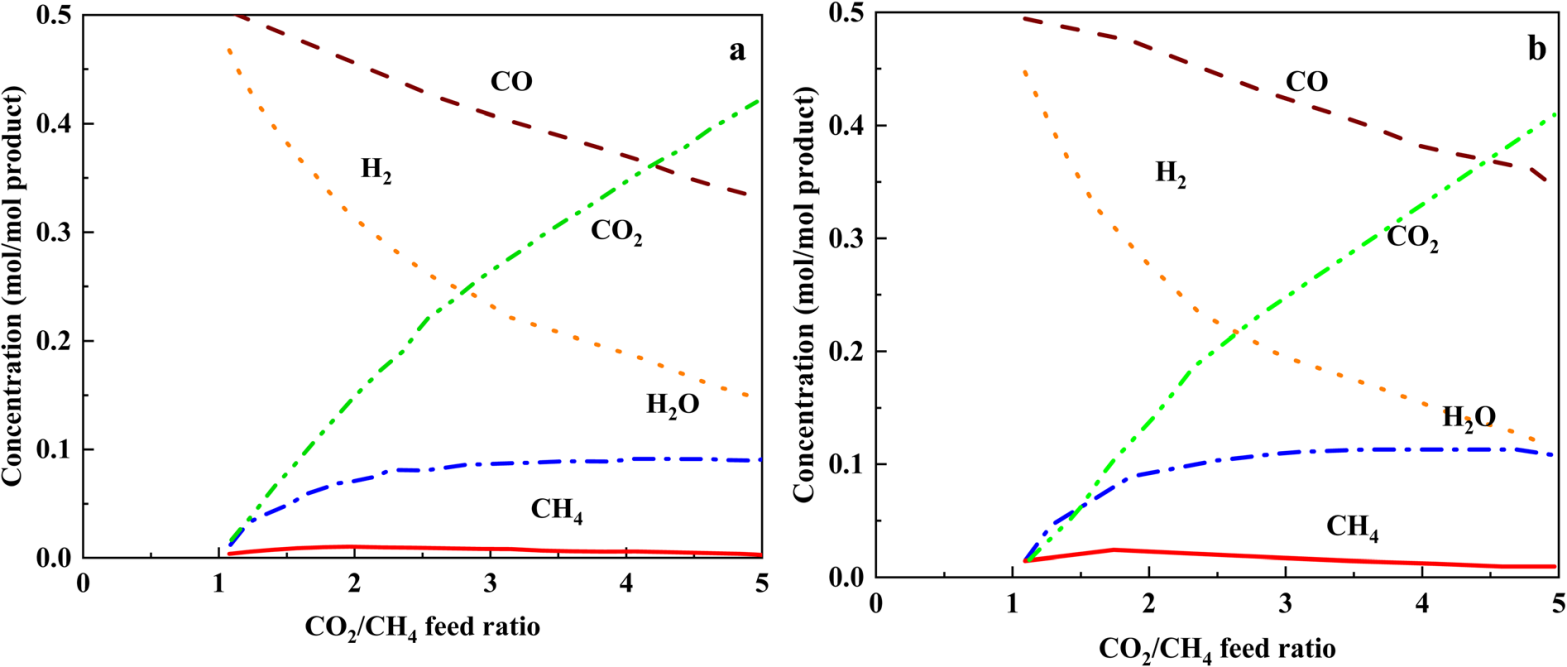
Equilibrium composition of CO_2_/CH_4_ system at (a) 1 atm and (b) 10 atm.^7^

Coke (C) is an undesirable byproduct as it impairs catalyst activity through physical blockage of reformer tubes, collapse of the catalyst support, encapsulation of metal crystals, and blockage of pores.^3^ There is general agreement that carbon formation occurs through the dissociation of CH_4_ (Rxn (4)) and the disproportionation of carbon monoxide Rxn (3)).^4,5^ However, two additional reactions are also considered contributors to coke formation: the hydrogenation of CO_2_ (Rxn (5)) and the hydrogenation of CO (Rxn (6))^6^ All of these reactions are exothermic except for the decomposition of methane (Rxn (4)). Fig. 1 illustrate the equilibrium composition of the reforming reaction at various temperatures and pressures (1 and 10 bar) with different feed ratios.^7,8^

**Fig. 2:**
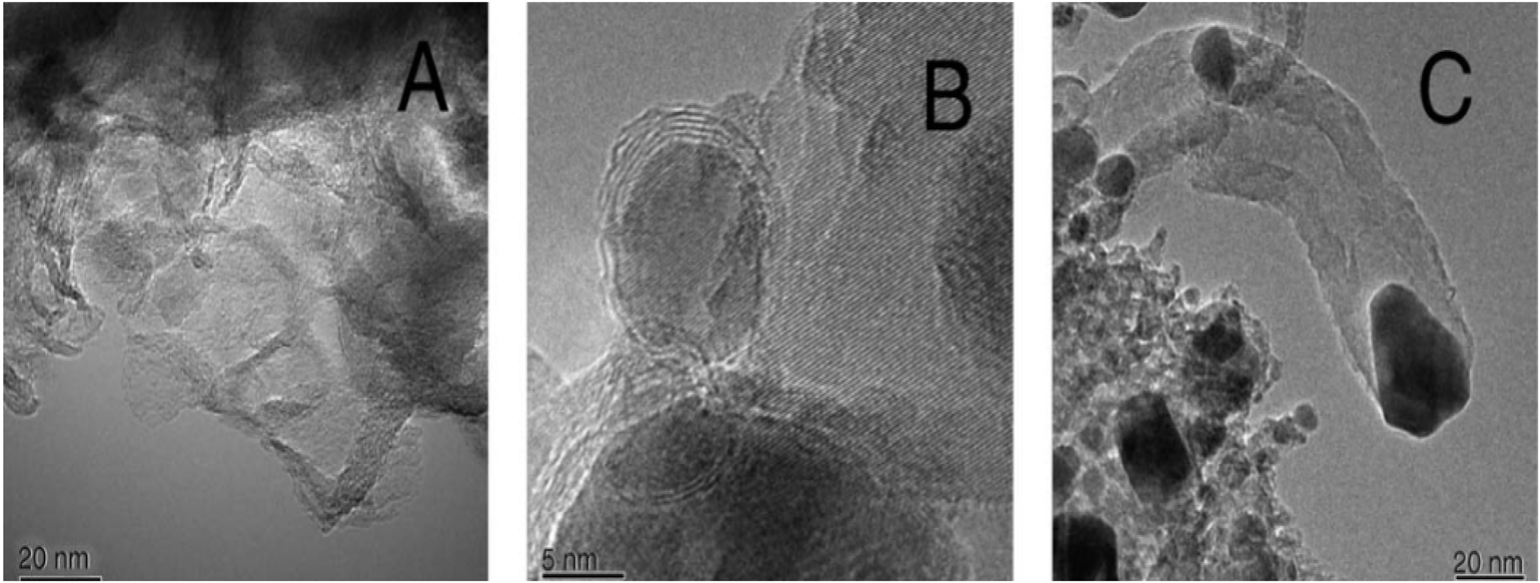
TEM images of the types of carbon; pyrolytic carbon on a MgAl_2_O_4_ carrier (A), encapsulating carbon (B), and whisker carbon (C) on Ni/MgAl_2_O_4_ reforming catalysts.^9^

The CO_2_ reforming of methane poses a risk of carbon formation, which can negatively impact catalyst activity. There are three types of carbon formation typically observed in a reformer: pyrolytic, encapsulating, and whisker carbon, as depicted by transmission electron microscopy in Fig. 2.^9^ The pyrolytic carbon (Fig. 2a) typically forms due to the exposure of higher hydrocarbons to high temperatures. The sintering or sulfur poisoning of the catalyst can result in reduced activity, causing higher hydrocarbons to reach elevated temperatures in the reformer.^10,11^ This type of carbon formation usually occurs at temperatures above 600 °C and is influenced by critical parameters such as high temperature, high void fraction, high pressure, and acidic catalysts.^12^ Carbon encapsulation occurs during the reforming of heavy hydrocarbon feeds with a high content of aromatic compounds (Fig. 2b). The high final boiling point and low temperatures of the hydrocarbon mixture contribute to an increased rate of encapsulating carbon formation.^9^ As shown in Fig. 2b Encapsulating carbon consists of a thin CH_*x*_ film that covers the Ni particles, leading to catalyst deactivation. Typically, this type of carbon formation occurs at temperatures below 500 °C.^12^ The final type of carbon formation is whisker carbon, which is the most critical type in the DRM reaction. Whisker carbon forms when hydrocarbons or CO react on one side of the Ni particle, leading to the growth of carbon whiskers. Simultaneously, graphitic carbon nucleates as carbon whiskers on the opposite side of the nickel particle, as illustrated in Fig. 2c.^9^ This type of carbon formation leads to the breakdown of the catalyst, an increase in pressure drop, and significant deactivation of the Ni surface. Whisker carbon typically forms at temperatures above 450 °C.^12^

### 2.2. Effect of H_2_O on DRM

**Fig. 3:**
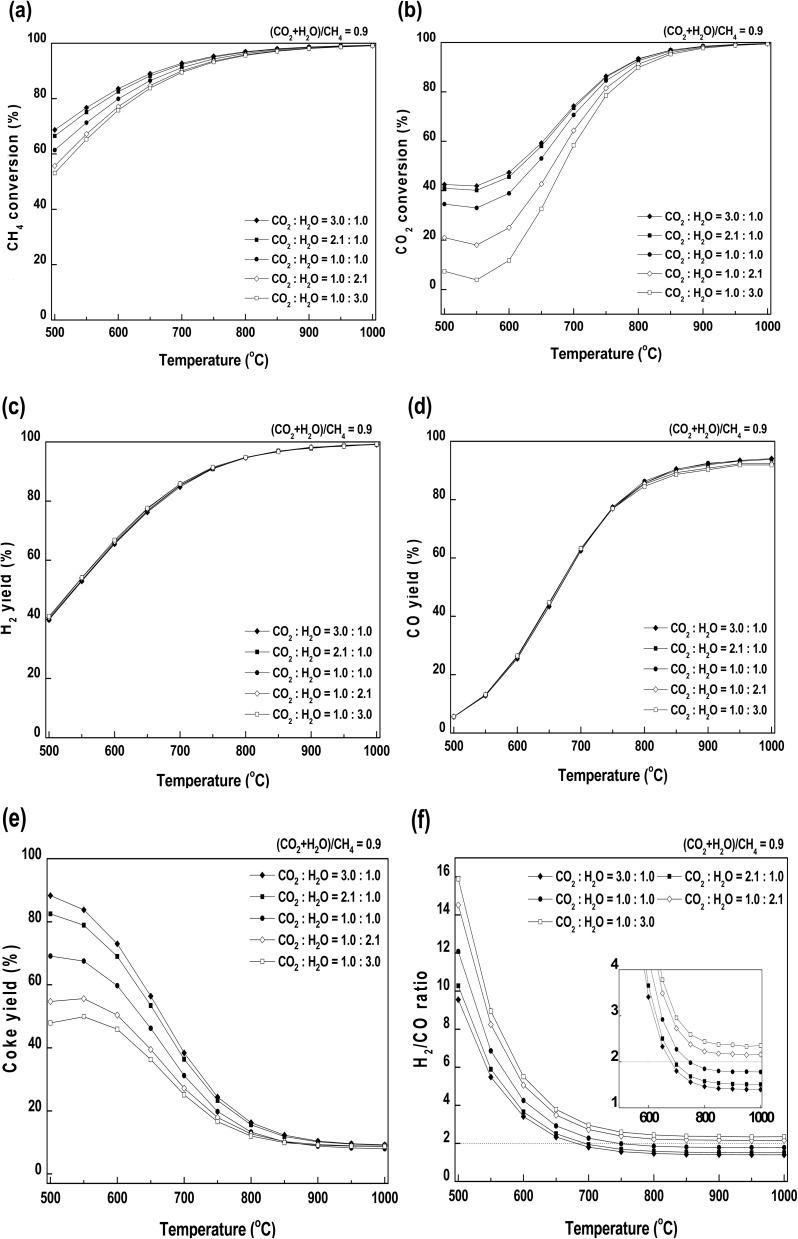
Effect of CO_2_ : H_2_O ratio as a function of temperature at a fixed (CO_2_ + H_2_O)/CH_4_ ratio of 0.9: (a) CH_4_ conversion, (b) CO_2_ conversion, (c) H_2_ yield, (d) CO yield, and (e) coke yield, and (f) H_2_/CO ratio.^12^

The influence of water addition on the DRM reaction was systematically investigated by Jang *et al.*^12^ across a temperature range of 500 to 1000 °C. The study employed a feed mixture of CH_4_ : CO_2_ : H_2_O : N_2_, using N_2_ as an internal standard, and was structured to probe three key variables: first, the total oxidizing capacity by varying the (CO_2_ + H_2_O)/CH_4_ ratio from 0.9 to 2.9 at a fixed CO_2_ : H_2_O ratio; second, the type of oxidant by adjusting the CO_2_ : H_2_O ratio from 3.0 : 1.0 to 1.0 : 3.0 at a fixed total oxidant-to-methane ratio; and third, the effect of pressure from 1.0 to 20 atm. As illustrated in Fig. 3a,^12^ CH_4_ conversion increased with temperature for all conditions, consistent with the endothermic nature of both steam and dry reforming reactions. A distinct crossover was observed at approximately 650 °C. Below this temperature, lower (CO_2_ + H_2_O)/CH_4_ ratios (0.9, 1.2, 1.4) yielded higher CH_4_ conversion, whereas above it, higher ratios (2.0, 2.9) proved more effective. Near-complete CH_4_ conversion was achieved above 850 °C for all but the lowest ratio (0.9), indicating that an insufficient supply of oxidizing agent acts as a limiting reactant. CO_2_ conversion exhibited a more complex profile (Fig. 3b),^12^ initially decreasing between 500–550 °C before rising at higher temperatures. The initial decline is attributed to the water–gas shift (WGS) reaction being more favorable than DRM at lower temperatures, effectively consuming produced CO and H_2_O to generate additional CO_2_. The subsequent increase above 550 °C confirms that the highly endothermic DRM becomes dominant. This biphasic trend is consistent with prior findings.^13^ The yield of H_2_ (Fig. 3c)^12^ increased with temperature for lower feed ratios due to contributions from SRM, DRM, and WGS, but peaked and began to decline above 800 °C, likely due to H_2_ consumption *via* the reverse water–gas shift (RWGS) reaction. Higher feed ratios resulted in diminished maximum H_2_ yields, as CH_4_ becomes the limiting reactant. Conversely, CO yield (Fig. 3d)^12^ increased monotonically with temperature. Below 650 °C, CO yield rose more rapidly for lower feed ratios, while above 750 °C, these lower ratios ultimately produced more CO, indicating that the total oxidant-to-methane ratio is a more significant factor for syngas yield than the specific CO_2_ : H_2_O ratio.^14^ A critical finding pertains to coke yield (Fig. 3e),^12^ which decreased with both increasing temperature and higher feed ratios. Coke deposition was effectively suppressed at temperatures above 650 °C for high feed ratios (2.0, 2.9) and approached zero at 750 °C for moderate ratios (1.2, 1.4). In contrast, the lowest ratio (0.9) exhibited significant coking even at high temperatures, demonstrating a direct link between insufficient oxidant and carbon formation. This confirms that an excess of oxidizing agent enhances CH_4_ conversion and suppresses coke.^14–16^ Furthermore, H_2_O was identified as more effective than CO_2_ in suppressing coke formation, as environments rich in CO_2_ promote CH_4_ activation pathways that lead to carbon, whereas H_2_O facilitates gasification. Finally, the H_2_/CO ratio (Fig. 3f)^12^ was high at lower temperatures, driven by H_2_ production from CH_4_ decomposition and CO consumption *via* the Boudouard and WGS reactions. The ratio stabilized as temperature increased. For a feed ratio of 0.9, it stabilized at 2.2, indicating complete consumption of the oxidant with residual CH_4_ forming H_2_ and coke. For ratios of 1.2 and 1.4, it approached the stoichiometric value of 2.0 for combined reforming above 850 °C. For higher ratios (2.0, 2.9), the H_2_/CO ratio remained above 2.0 between 750–800 °C due to the dominance of SRM, but dropped below 2.0 above 900 °C, signifying a shift towards the dominant DRM pathway and/or the increasing influence of the RWGS reaction. The data in Fig. 3e clearly demonstrate that increasing the H_2_O fraction in the feed reduces coke yield. This is because H_2_O gasifies carbon *via* the endothermic reaction C + H_2_O → CO + H_2_, which becomes favorable above 650 °C. Importantly, H_2_O is more effective than CO_2_ at suppressing coking under the same oxidant-to-methane ratio. Therefore, combined steam and dry reforming (also known as bi-reforming) represent a practical coke suppression strategy. Even small amounts of H_2_O (*e.g.*, H_2_O/CO_2_ molar ratio of 0.1–0.3) can significantly extend catalyst lifetime without excessively diluting the syngas product. Several recent pilot-scale studies and patents have adopted this approach for industrial DRM processes, as it allows operation at lower temperatures (600–700 °C) while maintaining low coke formation.

## 3. Ni-based catalysts for DRM

Nickel and noble metals (Rh, Ru, Pd, Pt, Ir) are extensively employed as active components in reforming catalysts, with noble metals demonstrating particularly promising performance in terms of conversion efficiency and selectivity during dry reforming. However, their high cost and limited availability render them impractical for widespread industrial application.^17^ Consequently, nickel has emerged as the most frequently used alternative due to its relative abundance and lower cost. A significant impediment to its use, however, is nickel's inherent propensity to catalyze carbon formation, which leads to rapid catalytic deactivation.^18^ This challenge has driven extensive research dedicated to improving the activity and stability of nickel-based catalysts. A primary strategy involves the incorporation of a second metal, which can improve the dispersion of the active phase and modify the interaction between nickel and the support material. The choice of support is equally critical; basic supports such as MgO, CaO, and Fe_2_O_3_,^19^ which possess fewer Lewis acid sites, and redox-active supports like CeO_2_ and ZrO_2_, known for their high oxygen mobility and thermal stability, have been shown to significantly enhance catalytic performance.^20^ These supports facilitate the adsorption and dissociation of CO_2_ on the catalyst surface, promoting the gasification of surface carbon and thereby mitigating its accumulation.^21^ The overarching goal for industrial commercialization of DRM is the development of affordable and efficient catalysts that simultaneously exhibit high activity and significant carbon resistance.^22^ To this end, research has broadly explored the effects of various support types, metallic promoters, and bi- or trimetallic combinations. Furthermore, catalyst preparation methods and pretreatment processes have been identified as crucial factors in determining the final structural properties, reduction behavior, and overall catalytic efficacy.^23^

### 3.1. Role of the support on catalyst activity

The catalytic performance in Dry Reforming of Methane (DRM) is profoundly influenced by the nature of the catalyst support, which governs metal–support interactions, active site dispersion, and reaction mechanisms. Research has extensively investigated DRM using various supported catalysts, including noble metals (Pt, Pd, Rh, Ru),^24,25^ and transition metals like Co and Fe.^26,27^ However, nickel (Ni) remains the most prevalent active metal for this reaction due to its cost-effectiveness and high intrinsic activity.^28,29^ A critical challenge associated with many Ni-based catalysts is their susceptibility to deactivation *via* carbon deposition, which progressively diminishes activity over time.^30^ Diverse support materials, including SiO_2_, La_2_O_3_, ZrO_2_, TiO_2_, CeO_2_, Al_2_O_3_, and MgO, have been explored to mitigate this issue, with studies focusing on evaluating activity, kinetics, and mechanistic steps to understand and reduce deactivation.^31–33^ The prevailing consensus in the literature describes the DRM mechanism as bi-functional. In this model, methane (CH_4_) is activated on the metallic sites (*e.g.*, Ni), while carbon dioxide (CO_2_) is activated on the support. The mechanism of CO_2_ activation is support-dependent: on acidic supports, it occurs through formate formation with surface hydroxyl groups, whereas on basic supports, it proceeds *via* the formation of oxy-carbonates.^34^ For catalysts supported on relatively inert materials like SiO_2_, a mono-functional mechanism is generally considered, where both reactants are activated solely on the metal particles. This often leads to significant carbon deposition from methane dehydrogenation, which hampers CO_2_ activation and reaction with carbon intermediates, resulting in rapid catalyst deactivation.^34,35^ The chemical interaction at the metal–support interface is crucial. For instance, the formation of a NiAl_2_O_4_ spinel phase during calcination enhances the Ni–Al_2_O_3_ interaction, leading to highly dispersed Ni nanoparticles (∼5 nm).^36^ This strong interaction not only reduces Ni agglomeration but also promotes reactant activation. Smaller Ni sites preferentially form amorphous carbon species that can be readily gasified during the reaction, resulting in low carbon deposition (*e.g.*, 3.8% over 20 hours at 700 °C) and high conversion rates (85.4% for CO_2_ and 77.6% for CH_4_) due to the abundance of accessible active sites and a large specific surface area.^36^

### 3.2. Metal–support interaction (MSI)

**Fig. 4:**
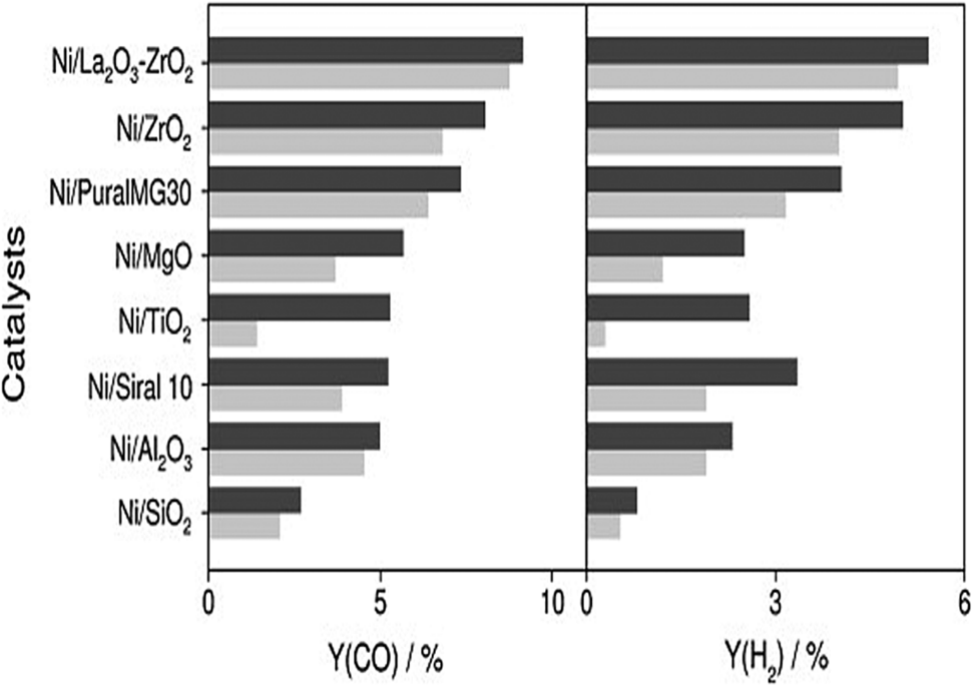
CO and H_2_ yields after first 10 h (black bars) and 100 h (gray bars) take place on DRM reaction at 400 °C and GHSV of 7200 mL h^−1^ g_cat_^−1^.^44^

The nature of the metal–support interaction (MSI) is a pivotal factor dictating the catalytic performance and stability in dry reforming of methane. Catalysts fabricated on inert supports such as SiO_2_ typically exhibit weaker MSI, which consequently results in lower stability and activity compared to their counterparts on mildly acidic (*e.g.*, Al_2_O_3_) or basic (*e.g.*, La_2_O_3_, CeO_2_, MgO) supports.^37–39^ While a weaker interaction can be advantageous in bimetallic systems by enhancing metal–metal synergy, strong MSI is generally sought for superior DRM catalysts. A prominent example is demonstrated with Ni supported on CeO_2_ and La-doped ceria, where strong interactions between nickel and lanthana, coupled with the surface oxygen vacancies inherent to ceria, foster the formation of Ce^3+^ sites and enhance Ni dispersion. This synergistic interaction was shown to yield high conversion rates of 96% for CH_4_ and 86.5% for CO_2_.^40^ The introduction of lanthana also generates intermediate and strong basic sites, which further augments CO_2_ interaction with the catalyst surface, thereby boosting overall performance. This enhanced reducibility arises from hydrogen spillover from Pt to NiO, which lowers the reduction temperature of Ni species. The resulting smaller, more uniformly dispersed Ni nanoparticles are less prone to sintering. Moreover, the Pt–Ni interface modifies the electronic structure of Ni, weakening the Ni–C bond strength and thereby suppressing the nucleation of graphitic carbon filaments. Together, these effects physically inhibit both coking and active phase sintering over extended time-on-stream.^41^ This highlights that not only the intrinsic nature of the support but also the specific metal–support combinations and the complex chemistry at their interfaces are paramount in DRM catalysis.^42^ Beyond directly participating in reactant activation through their acidic or basic character, supports also exert an indirect influence on the reaction mechanism by governing metal particle size and dispersion.^43^ A comparative study of various supported Ni catalysts (Ni/Al_2_O_3_, Ni/MgO, Ni/TiO_2_, Ni/SiO_2_, Ni/ZrO_2_, Ni/La_2_O_3_–ZrO_2_) (Fig. 4 and Table 1) at a low temperature of 400 °C revealed that catalysts incorporating Zr in the support, particularly Ni/La_2_O_3_–ZrO_2_, exhibited the highest initial activities, achieving CO and H_2_ yields near equilibrium and demonstrating the greatest stability.^44^ Notably, Ni/SiO_2_, despite possessing the highest specific surface area, showed the lowest initial H_2_ yield, emphasizing that strong MSI is more critical than high surface area alone. The superior stability of Ni/La_2_O_3_–ZrO_2_, with only a 9% reduction in H_2_ yield over 100 hours compared to a 20% loss for Ni/ZrO_2_, is attributed to an improved Ni–support interaction. This is likely a consequence of the partial encapsulation of NiO_*x*_ species within the mesoporous structure of the La_2_O_3_–ZrO_2_ support during preparation, which fosters the formation of strong chemical bonds and ensures a larger proportion of each Ni particle remains active and resistant to deactivation.^44–47^

**Table 1:** Mechanistic descriptors of selected supports for Ni-based DRM catalysts^a^

Support	Basicity	Oxygen vacancy concentration	Metal–support interaction strength	Carbon gasification rate (a.u.)	Dominant coke type	Ref.
CeO_2_	Moderate	High (Ce^4+^/Ce^3+^ redox)	Strong (SMSI)	High	Amorphous	40 and 48
ZrO_2_	Low	Moderate (Zr^4+/^Zr^3+^, minor)	Moderate	Moderate	Whisker	44 and 49
MgO	High	Very low	Strong (NiO/MgO solid)	Low (but high CO_2_ adsorption)	Encapsulating	50
Al_2_O_3_ (γ)	Low	None	Moderate (NiAl_2_O_4_ spinal at high T)	Low	Whisker + pyrolytic	36 and 51
La_2_O_3_	High	Low (but forms oxycarbonates)	Strong	Moderate	Amorphous (gasifiable)	31 and 52
SiO_2_	Very low	None	Weak	Very low	Wisker (sever)	35 and 53

a SMSI = strong metal–support interaction; a.u. = arbitrary units.

### 3.3. Effect promoter on catalytic activity

The strategic use of promoters is a critical avenue for enhancing the performance of nickel-based catalysts in dry reforming of methane. While noble metals such as Rh, Ir, Ru, and Pt are recognized for their high efficiency in DRM, their industrial application is constrained by scarcity and cost. Comparative studies, including work by Rostrup-Nielsen, have indicated that nickel can exhibit activity comparable to noble metals; however, its pronounced tendency for coke formation remains a significant disadvantage. To mitigate this, the incorporation of noble metal promoters like Pt has been shown to impart beneficial effects, primarily by preventing the sintering of nickel particles and thereby enhancing long-term catalyst stability.^54^ Sintering, as noted by Wu *et al.*, is another primary pathway for catalyst deactivation. Their investigation into Pt-promoted Ni/Al_2_O_3_ catalysts revealed that the structural interaction between Pt and Ni alters the catalyst's architecture, improving its resistance to sintering. This interaction fosters a competitive environment between the metals that facilitates the formation of more easily reducible Ni species. The concentration of the Pt promoter is a key variable, with lower concentrations leading to the strong formation of ionic oxides and consequently increasing CH_4_ conversion. Beyond Pt, the addition of other promoters such as potassium (K) and gold (Au) to Ni-based catalysts can create a synergistic interaction. This synergy promotes the reduction of NiO species and results in a smaller Ni particle size, which enhances the catalytic activity of bi- and trimetallic formulations. The composition of the active sites (*e.g.*, NiAu, NiPt, NiAuPt) directly influences the nature and amount of carbonaceous species accumulated on the catalyst surface, with a clear correlation observed between catalytic activity and carbon type. The most effective catalyst, such as the trimetallic NiAuPtAl system, represents an optimal balance of these parameters, characterized by a high proportion of less detrimental carbon nanotubes and a low quantity of total carbon deposit.^54,55^

### 3.4. Bi-trimetallic catalysts

The development of bi- and trimetallic catalysts represents a pragmatic and essential strategy for engineering highly coke-resistant nickel-based catalysts for dry reforming of methane, a area that has attracted substantial research interest in recent years.^56^ The combination of nickel with a second or third metal induces an alteration of its surface properties, often leading to enhanced catalytic activity through a phenomenon known as the synergistic effect. As demonstrated in a study by Elsayed *et al.*,^57^ supported bimetallic catalysts can exhibit both high activity and remarkable stability in the DRM reaction. In their investigation, catalysts comprising platinum (0.2–2 wt%), nickel (8 wt%), and magnesium (8 wt%) supported on a ceria-zirconia (Ce, Zr)O_2_ solid solution were synthesized. The incorporation of Pt into the Ni–Mg/(Ce, Zr)O_2_ system was found to significantly boost the low-temperature dry reforming activity compared to control catalysts lacking these components. A major factor contributing to this superior performance is the high dispersion of the active phases and the synergistic effects between platinum and the oxide matrix, which are correlated with the catalyst's reduction temperature and the population of its basic sites. Critically, the Pt/Ni–Mg/(Ce, Zr)O_2_ catalyst demonstrated efficient inhibition of carbon formation over an extended period of 100 hours on stream, outperforming catalysts with single Ni sites. This enhanced carbon resistance underscores the efficacy of multi-metallic formulations in mitigating the primary deactivation pathway in DRM. The performance of various Ni-based catalysts doped with different metal promoters is systematically summarized in Table 2, providing a comparative overview of their DRM reaction conditions, conversion percentages, H_2_/CO ratios, and synthesis methods.

**Table 2:** Performance of Ni-based catalysts doped with various metal species for DRM reactions

Catalysts	DRM Reaction conditions	CH_4_ conv.%	CO_2_ conv.%	H_2_/CO	Synthesis method	Ref.
Cs–Ni/FSA	750 °C, CH_4_/CO_2_/N_2_ = 1/1/2, 50 000 mL g^−1^ h^−1^, 1 bar, fixed bed reactor	95.0	90.0	0.48	Impregnation	58
5% Ni/10% Y-Z-3215	700 °C, CH_4_/CO_2_/N_2_ = 3/3/1, 42 000 mL g^−1^ h^−1^, 1 bar, fixed bed reactor	68.0	88.0	—	Impregnation	59
10% Ni/CeZrO_3_	700 °C, CH_4_/CO_2_/N_2_ = 1/1/1, 60 000 mL g^−1^ h^−1^, 1 bar, fixed bed reactor	74.0	55.0	1.40	Impregnation	60
Ni–PD/SiO_2_	700 °C, CH_4_/CO_2_ = 1/1, 90 000 mL g^−1^ h^−1^, 1 bar, fixed bed reactor	84.0	78.0	0.98	Impregnation	61
0.5RuNi/MgAl_2_O_4_	750 °C, CH_4_/CO_2_/N_2_ = 35/35/30, 110 mL g^−1^ h^−1^, 1 bar, fixed bed reactor	81.8	75.0	0.90	Impregnation	62
5% Ni–1% Mg/MCM-41	700 °C, CH_4_/CO_2_/N_2_ = 1/1/1, 60 000 mL g^−1^ h^−1^, 1 bar, fixed bed reactor	68.8	57.0	0.70	Impregnation	63
Ni–CZ/CFF	800 °C, CH_4_/CO_2_ = 1/1, 48 000 mL g^−1^ h^−1^, 1 bar, fixed bed reactor	59.8	92.0	1.18	Impregnation	64
NiLa/SiO_2_	750 °C, CH_4_/CO_2_/Ar = 4/10/85, 5000 h^−1^, 1 bar, fixed bed reactor	64.4	69.5	0.85	Impregnation	52
20% Ni/Si–MCM-41-TEOS	800 °C, CH_4_/CO_2_ = 1/1, 30 000 mL g^−1^ h^−1^, 1 bar, fixed bed reactor	93.0	95.0	1.58	Impregnation	65
10Ni/DFSAB-15	794 °C, CH_4_/CO_2_ = 50/50, 23 815 mL g^−1^ h^−1^, 1 bar, fixed bed reactor	93.5	95.7	0.98	Impregnation	66
16.5% Ni/16.5% Co/Al_2_O_3_	700 °C, CH_4_/CO_2_/Ni = 10/10/80, 15 090 mL g^−1^ h^−1^, 1 bar, fixed bed reactor	95.8	85.1	0.85	Co-precipitation	67
20% Ni/3% MgO–Al_2_O_3_	700 °C, CH_4_/CO_2_ = 1/1, 12 000 mL g^−1^ h^−1^, 1 bar, fixed bed reactor	72.0	77.0	0.83	Impregnation	51
CeO_2_–Ni	700 °C, CH_4_/CO_2_/Ar = 30/30/140, 180 000 mL g^−1^ h^−1^, 1 bar, fixed bed reactor	79.2	83.0	0.98	Impregnation	48
Ni_0.1_Mg_0.9_O	700 °C, CH_4_/CO_2_ = 4/1, 14 000 mL g^−1^ h^−1^, 1 bar, fixed bed reactor	96.6	41.8	0.46	Co-precipitation	50
Ni–CaO–ZrO_2_	850 °C, CH_4_/CO_2_ = 1/1.2, 158 000 mL g^−1^ h^−1^, 1 bar, fixed bed reactor	91.5	80.0	0.85	Co-precipitation	68
1% Ni–2%/ZSM5	800 °C, CH_4_/CO_2_/Ar = 20/20/60, 60 000 mL g^−1^ h^−1^, 1 bar, fixed bed reactor	80.0	86.0	0.98	Impregnation	69
HNiZr_3_	750 °C, CH_4_/CO_2_/Ar = 1/1/8, 20 000 mL g^−1^ h^−1^, 1 bar, fixed bed reactor	88.0	90.0	0.88	Co-precipitation	49
Ni–Al-MCM-41	750 °C, CH_4_/CO_2_ = 1/1, 150 000 mL h^−1^ g^−1^, 1 bar, fixed bed reactor	92.0	95.0	1.00	Impregnation	70
Ni/10% MgO–beta	800 °C, CH_4_/CO_2_ = 1/1, 60 000 mL g^−1^ h^−1^, 1 bar, fixed bed reactor	95.0	97.0	1.00	Impregnation	71
Ni–Y/KIT-6	750 °C, CH_4_/CO_2_/Ar = 1/1/8, 20 000 mL g^−1^ h^−1^, 1 bar, quartz reactor	85.0	89.0	0.80	Impregnation	72
1% Ni–2% Co–ZSM-5	700 °C, CH_4_/CO_2_/N_2_ = 1/1/3, 60 000 mL g^−1^ h^−1^, 1 bar, quartz reactor	60.0	65.0	0.82	Wet impregnation	73

### 3.5. Influence of synthesis methods on coke resistance

The preparation method critically determines Ni particle size, dispersion, and metal–support interaction all of which directly affect coke formation. Wet impregnation is simple but often yields broad Ni particle size distributions, leading to uneven coke deposition. Co-precipitation can produce more uniform nanoparticles (*e.g.*, Ni–MgO solid solutions) with strong metal–support interaction, reducing sintering and whisker carbon formation. However, co-precipitation requires careful pH control to avoid phase separation. Sol–gel methods allow high Ni dispersion but are more complex and expensive. The choice of Ni precursor also matters. Nickel nitrate decomposes at lower temperatures but leaves residual NO_*x*_ that can affect support acidity; nickel acetate produces larger particles but fewer impurities. Calcination temperature is critical: too low (below 400 °C) leaves NiO poorly reducible; too high (above 800 °C on Al_2_O_3_) forms inactive nickel aluminate (NiAl_2_O_4_), which reduces activity. Reduction temperature and atmosphere (H_2_ concentration, flow rate) influence final Ni crystallite size smaller particles (5–10 nm) are more resistant to whisker carbon because they favor amorphous carbon that is easily gasified. In summary, optimal coke resistance is achieved by synthesis protocols that yield small, well-dispersed Ni particles with strong metal–support interaction. Readers are referred to primary literature for detailed procedures on each method.

### 3.6. Contradictory reports and mechanistic debates

Despite extensive research, several key disagreements persist in the DRM literature. Resolving these contradictions is essential for rational catalyst design.

#### 3.6.1 Dominant coke source

Some studies identify methane cracking (CH_4_ → 2H_2_ + C) as the primary coke pathway at temperatures above 650 °C, while others argue that the Boudouard reaction (2CO → CO_2_ + C) dominates, particularly at lower temperatures (<700 °C). Giehr *et al.*^74^ used isotopic labeling to show that methane cracking contributes >80% of carbon deposits under typical DRM conditions (700 °C, 1 bar, CH_4_/CO_2_ = 1). In contrast, Nikoo and Amin^6^ concluded that the Boudouard reaction becomes significant only when CO partial pressure exceeds 0.5 bar. This discrepancy likely arises from differences in catalyst Ni particle size (larger particles favor CH_4_ cracking) and support basicity (basic supports enhance CO_2_ adsorption, shifting the equilibrium away from Boudouard).

#### 3.6.2 Pt *vs.* Ru promotion

Pt and Ru are both effective noble metal promoters, but their mechanisms differ. Pt enhances Ni reducibility *via* hydrogen spillover and improves sintering resistance, but Pt itself can coke under reducing conditions. Ru, on the other hand, exhibits superior intrinsic activity for CO_2_ dissociation and carbon gasification, but Ru is more prone to oxidation in the presence of trace O_2_. Álvarez Moreno *et al.*^62^ compared Pt–Ni and Ru–Ni on MgAl_2_O_4_ and found that Ru–Ni gave higher initial activity but Pt–Ni showed better stability beyond 50 h. The trade-off between activity and stability remains unresolved and likely depends on reaction conditions and support choice.

#### 3.6.3 MgO as a support

MgO is widely praised for its basicity and strong metal–support interaction. However, several studies report that Ni/MgO suffers from severe Ni sintering above 750 °C due to the formation of mobile Ni(OH)_2_ species under the H_2_O-rich micro-environment created by RWGS. Zanganeh *et al.*^50^ found that only Ni_*x*_Mg_1−*x*_O solid solutions (with Ni content < 20 mol%) remain stable, whereas higher Ni loadings lead to rapid deactivation. This nuance is often overlooked in descriptive summaries.

## 4. Understanding deactivation, regeneration and reclamation of deactivated catalyst

### 4.1. Deactivation of metal catalysts

Catalyst deactivation in DRM is a multifaceted phenomenon, but coking (carbon deposition) and sintering are the primary causes for Ni-based systems. While sintering reduces active surface area, coking physically and chemically disrupts the catalyst.

#### 4.1.1 Thermodynamics of coke formation

Deactivation of Ni-based DRM catalysts occurs *via* several mechanisms, but coking and sintering are the dominant pathways. This section analyzes each mechanism with a focus on how catalyst design can mitigate them. Understanding the formation and growth of coke, which is a primary deactivation pathway for catalysts used in DRM, poses a significant challenge. Coke can form through various reactions, including both catalytic and non-catalytic pathways. In addition to the generation of coke precursors and coke *via* non-catalytic gas-phase reactions, which will be discussed separately, three main pathways are considered relevant for coke formation under DRM conditions. The Boudouard reaction, which produces carbon through CO disproportionation (eqn (7)), is generally more favorable at lower temperatures.

7 2CO → CO_2_ + C Δ*H*_298_ = −171 kJ mol^−1^

The reduction of CO by H_2_ to form carbon and water is more favorable at low temperatures; however, it is generally of low significance due to its consistently higher Gibbs free energy, except in gas mixtures with high hydrogen partial pressures.^75^

8 CO + H_2_ → H_2_O + C Δ*H*_298_ = −131 kJ mol^−1^

Coke formation resulting from methane decomposition, including methane cracking *via* radical pathways and subsequent methane decarbonation, is thermodynamically favored at high temperatures.

9 CH_4_ → 2H_2_ + C Δ*H*_298_ = 75 kJ mol^−1^

**Fig. 5:**
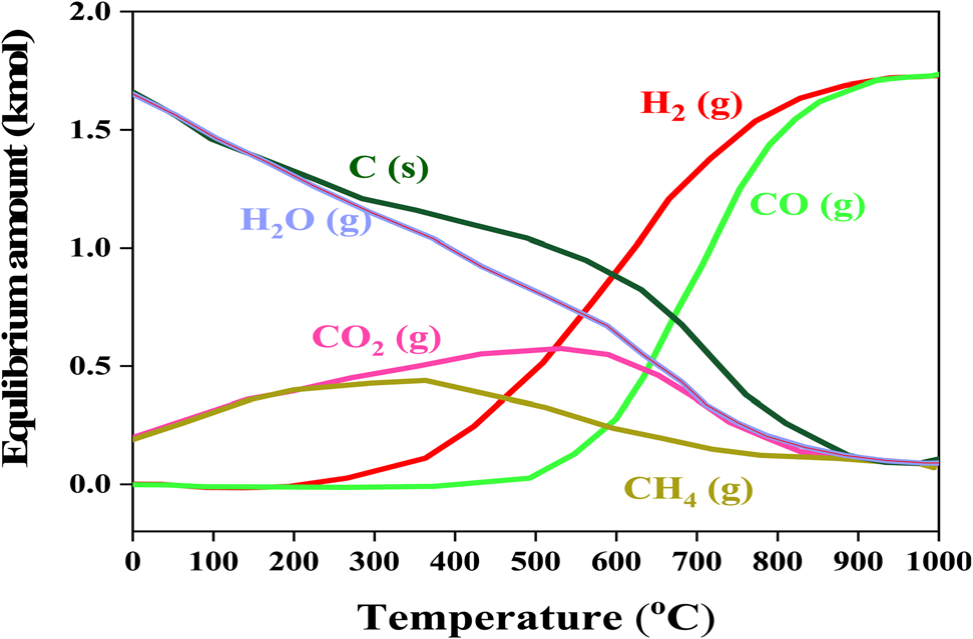
Thermodynamic equilibrium plots for DRM at 1 atm, 0–1000 °C, and a CO_2_/CH_4_ feed ratio of 1 : 1. These equilibrium calculations were carried out using the Gibbs free energy minimization algorithm on HSC Chemistry 7.1 software.^82^

**Fig. 6:**
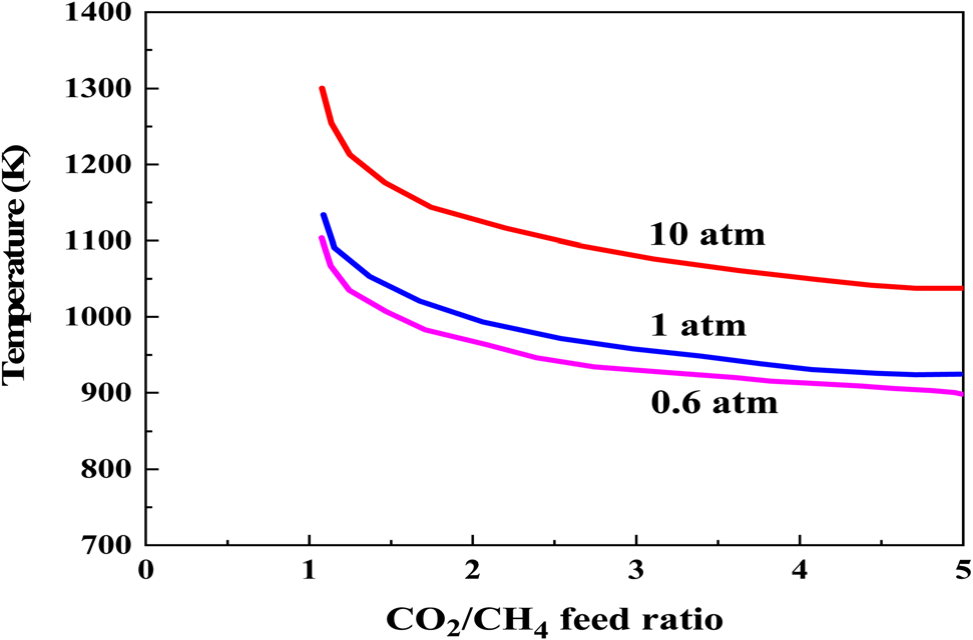
Thermodynamic evaluation of total pressures, temperatures, and feed ratios for carbon production.^7^

**Fig. 7:**
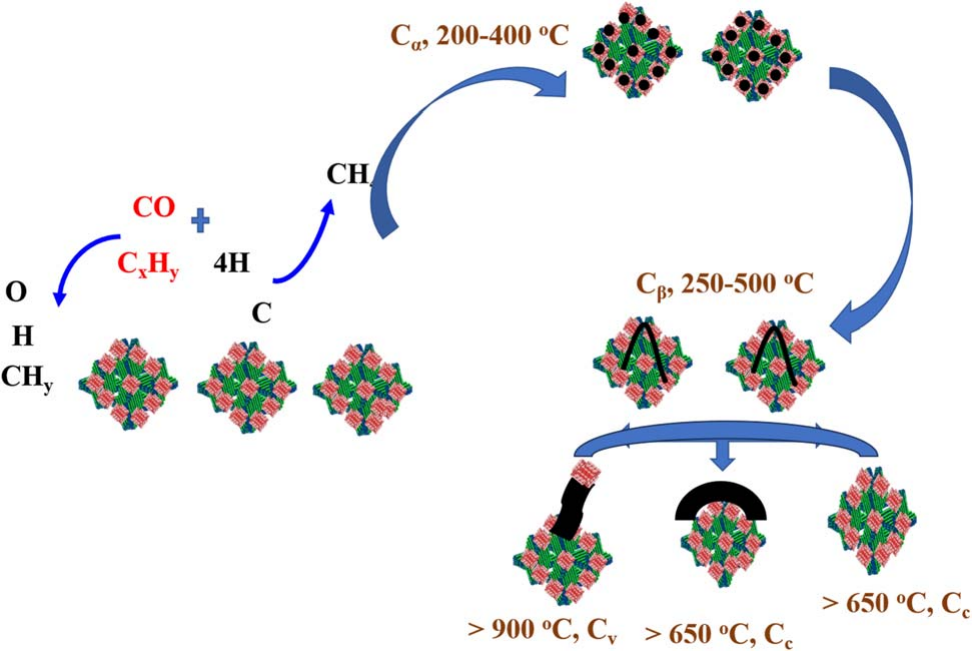
Carbon production mechanism at the catalyst surface.

**Fig. 8:**
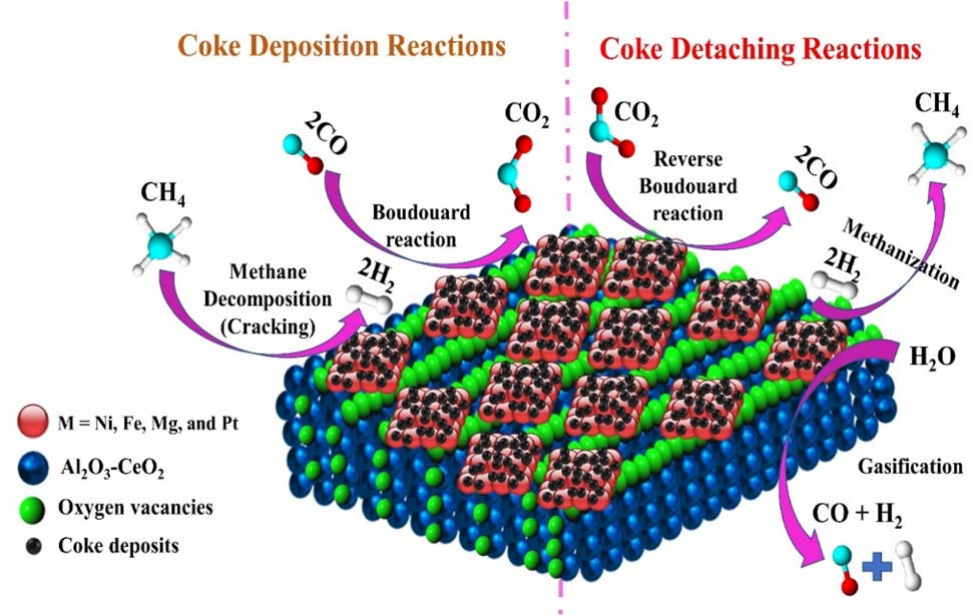
Overview of coke forming reactions.

These reactions alter the composition of syngas and lead to catalyst deactivation. A significant challenge in dry reforming is the rapid deactivation of the catalyst by coke formation when steam is not used. Thermodynamically, the endothermic reaction (methane dissociation) occurs at temperatures above 557 °C, while exothermic reactions (Boudouard reaction) occur below 700 °C. Consequently, the maximum carbon deposition is observed in the temperature range of 557–700 °C,^76,77^ as indicated by the equilibrium data for the DRM reaction (Fig. 5).^7^ Each total pressure value has a specific temperature above which no carbon formation occurs relative to the CH_4_/CO_2_ feed ratio, as illustrated in Fig. 6.^14^ At atmospheric pressure, carbon formation will occur below 870 °C. Fig. 5 and 6 indicate that the optimal temperature range for the DRM process is 643–1027 °C and the pressure close to atmospheric.^78,79^ Various types of carbon species, including C_α_, C_β_, C_γ_, C_v_, and C_c_, are deposited during the DRM process (for nomenclature see Table 3).^80^ CO and CH_4_ dissociate at the catalyst surface to produce Cα, an adsorbed atomic carbon; C_α_ can then transform into C_β_, a polymeric carbon, or amorphous films, which further react to form C_γ_, C_v_, and C_c_.^81^ These transformations are illustrated in Fig. 5 (temperature dependence) and Fig. 7 (surface mechanism). Some carbon forms are more detrimental than others: C_β_ below 300–400 °C and C_c_ above 650 °C encapsulate Ni particles, while whisker carbon (C_v_) at 300–1000 °C lifts Ni off the support, causing reactor plugging.^91–93^ Carbon removal (gasification) can occur *via* the reverse of reactions (8) and (9) or the reverse Boudouard reaction (reverse of eqn (7)). Giehr *et al.*^74^ found that methane cracking is the main coke source under typical DRM conditions (Fig. 8).

**Table 3:** Details of the various carbon species generated on the catalyst surface^80^

Carbon structure	Designation	Temperature range (°C)
Adsorbed, atomic carbon (surface carbide)	C_α_	200–400
Polymers, amorphous films	C_β_	250–500
Ni carbide (bulk)	C_γ_	150–250
Vermicular filaments or whiskers	C_v_	300–1000
Graphite (crystalline) platelet films	C_c_	500–550

#### 4.1.2 Mechanism of catalyst deactivation

In industrial processes involving organic compounds and solid catalysts, catalyst deactivation is an inevitable phenomenon that significantly impacts process efficiency and economics. The mechanisms of deactivation are multifaceted and typically arise from a combination of factors rather than a single cause. These mechanisms are systematically categorized into three primary groups: mechanical, thermal, and chemical, with the latter encompassing several specific pathways. The principal chemical mechanisms contributing to catalyst degradation include: (i) poisoning, (ii) coking and fouling, (iii) sintering, (iv) component volatilization, (v) formation of inactive compounds, (vi) phase transformation, and (vii) mechanical failure or attrition.

##### 4.1.2.1. Poisoning

Poisoning constitutes a prevalent mechanism of catalyst deactivation, characterized by the strong, irreversible chemisorption of species that are not participants in the desired reaction pathway. This process effectively “blocks” the active sites on the catalyst surface, preventing reactant access and adsorption. The role of a specific compound as a poison is contingent upon both the nature of the catalytic process and the comparative adsorption strength of the poison relative to the reactants; poisoning occurs when the affinity of the poison for the active site surpasses that of the reaction species. Beyond the physical obstruction of sites, poisoning can induce more profound detrimental effects. These include electronic modification of adjacent metal atoms, which impairs their ability to adsorb and dissociate reactants; significant restructuring or rearrangement of the catalyst surface, altering its intrinsic catalytic properties; and interference with the surface diffusion of adsorbed reactants, thereby hindering the reaction sequence. In severe cases, poisoning can also lead to the formation of new, inactive chemical compounds or provoke structural changes in the catalyst itself, leading to a permanent loss of function.^83,84^

##### 4.1.2.2. Fouling/coking deposition

**Fig. 9:**
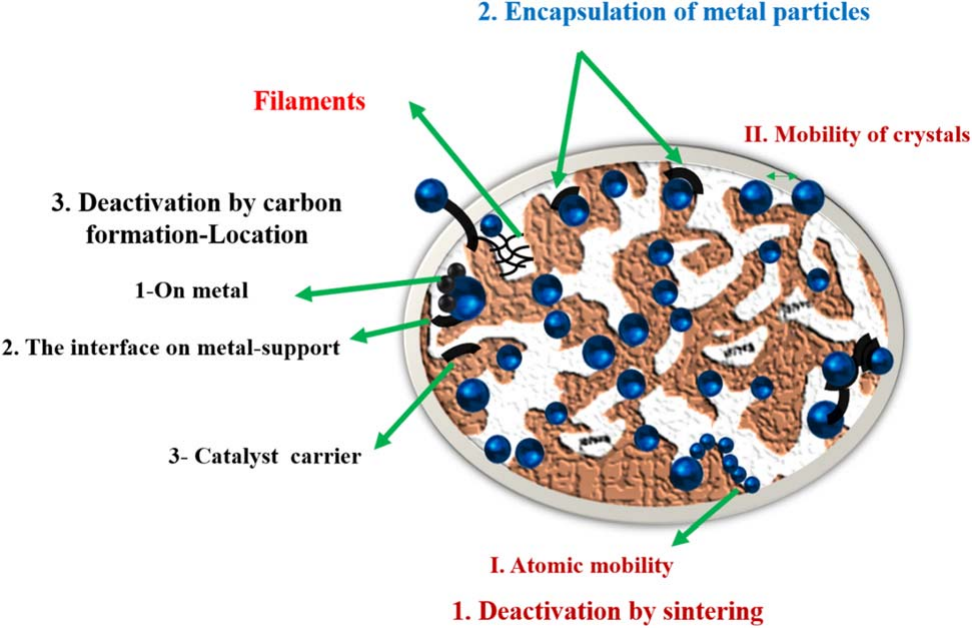
Schematic illustration of major deactivation mechanisms in Ni-based DRM catalysts. The image shows: (1) sintering particle migration and coalescence (large clusters) and Ostwald ripening (small particle shrinking, large particle growing); (2) coking carbon encapsulation of Ni particles (dark layer around metal) and pore blockage (carbon filling a support pore); (3) poisoning a foreign atom (*e.g.*, S, Cl) strongly adsorbed on a Ni active site, blocking reactant access.

Fouling and coking represent a primary deactivation mechanism in heterogeneous catalysis, characterized by the physical deposition of species from the fluid phase onto the catalyst surface.^85^ While “fouling” can encompass various types of deposits, “coking” specifically refers to the formation of carbonaceous materials. This process leads to a loss of catalytic activity through the physical blockage of active sites and/or the constriction of catalyst pores, thereby impeding reactant access. In more severe manifestations, it can also cause the disintegration of catalyst particles and ultimately lead to reactor blockage. The deleterious effects of carbon deposition, as illustrated in Fig. 9, are multifaceted: (i) the deposited coke either chemisorbs strongly to the metal particle or forms a multi-layer physically adsorbed film, which restricts the diffusion of reactants to the active sites; (ii) complete encapsulation of the metal particle by carbon results in its total deactivation; (iii) the obstruction of pores within the support material prevents reactants from reaching the internal active sites; and (iv) the formation and growth of carbon filaments can exert sufficient mechanical stress to fracture the support material, potentially leading to a heightened pressure drop and plugging of the reactor.^83,86,87^

##### 4.1.2.3. Sintering

Sintering is a thermally induced deactivation mechanism that affects both supported and unsupported catalysts, leading to a significant reduction in the active surface area through the growth of metal crystallites and the degradation of the support structure. This phenomenon is primarily driven by exposure to harsh reaction conditions or elevated pre-treatment temperatures, although the reaction atmosphere and the strength of interactions between catalyst components also play critical roles. The mechanisms by which metal particles increase in size, as depicted in Fig. 9, primarily involve: (i) atomic migration, where individual metal atoms detach from smaller crystallites, diffuse across the support surface, and are captured by larger particles a process known as Ostwald ripening; (ii) crystallite migration, where entire particles migrate and coalesce upon collision to form larger aggregates; and (iii) particle spreading and subsequent splitting. The decay in catalytic activity from sintering results from two principal outcomes: the growth of crystallites in the active phase, which directly reduces the available catalytic surface area, and the collapse of the support material and its porous network, which diminishes the area available for metal dispersion and limits substrate access to active sites. The support itself is also susceptible to sintering through various processes, including surface diffusion, solid-state diffusion, grain boundary diffusion, the evaporation and condensation of volatile atoms or molecules, and phase transformations.^83,86,88^

##### 4.1.2.4. Gas/vapor–solid and solid-state reactions

Another chemical pathway contributing to catalyst deactivation involves the reaction between the vapor phase and the catalyst surface, resulting in the formation of inactive phases rather than strongly adsorbed species, thereby reducing catalyst activity. These reactions typically occur at high temperatures and can be readily detected using X-ray diffraction (XRD). Examples include the reaction of the active nickel phase with common supports like Al_2_O_3_ or SiO_2_ to form nickel aluminates or silicates, the formation of oxides with steam when cobalt or iron are used,^89^ or the oxidation, sulfidation, or carbidation of the metal active phase, all of which lead to a loss of catalytic activity.^84^ Co and Fe are much explored less frequently than Ni on DRM catalysts but investigated as a Ni substitutes or promoters. Under DRM conditions (*i.e.*705–900 °C, typically with trace H_2_O (as an impurity or co-feed) present in the inlet CO_2_ and CH_4_ feeds), cobalt can be oxidized to CoO or Co_3_O_4_ by steam according to reactions like: Co + H_2_O → CoO + H_2_ which results in the formation of an inactive oxide layer. Likewise, in oxidizing local conditions iron might become Fe_2_O_3_. Sulfidation, although more relevant to feeds containing H_2_S (biogas or industrial tail gas), can also indeed take place during DRM where the presence of sulfur compounds allows nickel and iron to rapidly give rise to bulk sulfides Ni_3_S_2_ or FeS with negligible activity for methane activation. So, these vapor-solid reactions pertain directly to DRM since the feed is not pure.^84,89^

##### 4.1.2.5. Phase transformation

Phase transformation constitutes a significant pathway for catalyst deactivation, primarily resulting from the thermal degradation of the support material, which adversely impacts its physical and chemical properties. This process involves the irreversible transition of the support from a high-surface-area, metastable phase to a more thermodynamically stable, low-surface-area phase. A canonical example is the transition of γ-Al_2_O_3_ support through intermediate phases such as δ-Al_2_O_3_ and θ-Al_2_O_3_, culminating in the formation of α-Al_2_O_3_, the most stable crystalline phase. This series of transformations precipitates a substantial decrease in the specific surface area of the supported catalyst, thereby directly reducing the available area for active metal dispersion and leading to a consequential decline in catalytic activity. This thermal degradation process is frequently described in the literature as support sintering.^83^ Strategies to mitigate this detrimental phase change have been explored, including the addition of small amounts of silica, which acts as a structural stabilizer to inhibit the transformation to α-Al_2_O_3_ and help preserve surface area.^90,91^

##### 4.1.2.6. Particle attrition

Particle attrition represents a critical mode of mechanical failure in heterogeneous catalysts, leading to the physical degradation of the catalyst bed and operational inefficiencies. This deactivation pathway can result from several factors: (i) the crushing of catalyst granules, pellets, or monolithic structures during the loading procedure; (ii) the gradual attrition or reduction in catalyst particle size, resulting in the generation of fine powders, a phenomenon particularly prevalent in fluidized bed reactors; and (iii) surface erosion induced by high fluid velocities passing through the catalyst bed.^84^ The consequences of such mechanical failure are substantial, often causing reactor plugging or flow channeling, which in turn leads to an increased pressure drop across the reactor and non-uniform bed performance that compromises conversion and selectivity. Thermal stresses and carbon deposition can exacerbate other degradation mechanisms, further accelerating attrition. To mitigate these issues, several preventive strategies have been proposed in the literature, including: (i) enhancing the intrinsic mechanical strength through advanced catalyst synthesis techniques; (ii) incorporating binders to improve the overall strength and toughness of the catalyst bodies; (iii) coating catalyst aggregates with porous yet mechanically robust materials to provide a protective shell; and (iv) inducing beneficial compressive stresses within the catalyst particles *via* chemical or thermal tempering processes.^83^

### 4.2. Regeneration

The regeneration of deactivated catalysts represents a critical process in catalytic systems, balancing economic considerations with environmental impacts. Catalysts in packed-bed reactors undergo substantial mechanical stress from thermal cycling during startup and shutdown operations, in addition to chemical exposure from corrosive reaction media.^92,93^ Regeneration offers a sustainable pathway for reusing catalysts in their original processes or repurposing them for other applications; for example, carbon-deposited catalysts can be utilized in electrode manufacturing. Carbon deposition, a predominant deactivation mechanism, is principally reversible through gasification processes. Research by Son *et al.*^94^ demonstrated that pretreatment of Ni/γ-Al_2_O_3_ catalysts with steam at 850 °C significantly enhanced stability, achieving near-equilibrium conversions (98.3% for CH_4_ and 82.4% for CO_2_) with an optimal H_2_/CO ratio of 2.01 during combined reforming, while maintaining operational stability over 200 hours. The steam-treated catalyst exhibited merely 3.6% coke deposition compared to 15.4% in conventional catalysts, as steam pretreatment effectively removed unstable aluminum species responsible for initial coke formation. Standard regeneration typically involves coke combustion with air, generating CO_2_ through a highly exothermic reaction. For complex carbon deposits, solvent extraction provides an alternative cleaning method. However, when regeneration proves economically unfeasible, catalyst disposal becomes necessary. Investigations by Steib^95^ on Ni/ZrO_2_ catalysts revealed that regeneration using CO_2_ rather than O_2_ significantly enhances catalytic activity through dynamic restructuring of the Ni–ZrO_2_ interface. In this mechanism, the ZrO_2−*x*_ support catalytically activates CO_2_, transferring oxygen to nickel sites and releasing CO *via* carbonate decomposition establishing a more efficient pathway than direct CO_2_ dissociation on metallic nickel. While O_2_ regeneration produces CO_2_ and causes metal sintering through exothermic oxidation, CO_2_ regeneration operates *via* the endothermic reverse Boudouard reaction, simultaneously removing carbon deposits while minimizing thermal degradation and preserving catalyst microstructure, as confirmed by Extended X-ray absorption fine structure (EXAFS) analysis. Emerging regeneration methods include non-thermal plasma-assisted coke removal, which operates at lower temperatures and avoids thermal sintering. Additionally, regeneration using CO_2_ instead of O_2_ has been shown to preserve catalyst microstructure because it proceeds *via* the endothermic reverse Boudouard reaction, whereas O_2_ regeneration causes hot spots and metal sintering. Future work should explore cyclic regeneration protocols that combine mild oxidation with hydrogen reduction to fully restore activity.

### 4.3. Limitations of state-of-the-art Ni-based DRM catalysts

Despite significant advances, no current Ni-based catalyst meets all industrial requirements simultaneously. Below we summarize the key limitations.

#### 4.3.1 Sintering-activity trade-off

Very small Ni nanoparticles (<5 nm) exhibit high activity but sinter rapidly above 700 °C. Larger particles (10–20 nm) are more stable but have lower intrinsic activity and promote whisker carbon formation. This trade-off remains unresolved.

#### 4.3.2 Pressure gap

Nearly all mechanistic studies are conducted at 1 bar, but industrial DRM processes would operate at 5–20 bar to reduce reactor volume. At elevated pressure, coke formation is thermodynamically more favorable (see Fig. 6), and many catalysts that perform well at 1 bar fail under pressure. Only a handful of studies have tested catalysts above 10 bar, and those show drastically reduced stability.

#### 4.3.3 Poisoning sensitivity

Real DRM feeds (biogas, flue gas, or natural gas with CO_2_) contain trace impurities such as H_2_S (0.1–100 ppm), NH_3_, and higher hydrocarbons. Ni-based catalysts are extremely sensitive to H_2_S, with deactivation occurring even at <0.1 ppm. Noble metal promoters (Pt, Ru) slightly improve sulfur tolerance, but not sufficiently for industrial applications. This area remains largely unexplored.

#### 4.3.4 Regeneration inefficiency

Oxidative regeneration (air or O_2_) removes coke but causes irreversible Ni sintering due to the exothermic nature of carbon combustion (Δ*H* = −393 kJ mol^−1^ C^−1^). Local hot spots can raise temperatures >100 °C above setpoint. CO_2_ regeneration (reverse Boudouard) is milder but slower and leaves some refractory carbon untouched. No regeneration protocol has been demonstrated to fully restore initial activity over multiple cycles.

#### 4.3.5 Scalability of advanced synthesis

Laboratory-scale successes (*e.g.*, core–shell, yolk–shell, and MOF-derived catalysts) rely on multi-step syntheses that are difficult and expensive to scale. For example, the Ni@m–SiO_2_ yolk–shell catalyst described by Zhao *et al.*^23^ shows excellent coke resistance but requires template removal, etching, and multiple calcination steps, making it impractical for ton-scale production.

#### 4.3.6 Quantitative gap to industrial targets

Table 5 (below) compares reported stability durations and coke rates to industrial requirements. Most laboratory studies report stability for 50–200 h, whereas industrial reformers require >8000 h (≈1 year) on stream. Coke deposition rates below 1 mg g^−1^ h^−1^ are considered acceptable for laboratory prototypes, but industrial catalysts require <0.1 mg g^−1^ h^−1^ to avoid pressure drop issues over the catalyst lifetime.

### 4.4. Reclamation

The management of spent hydroprocessing catalysts has emerged as a significant environmental challenge, with the volume of this hazardous solid waste escalating in parallel with the expansion of global hydroprocessing capacity mandated for low-sulfur fuel production. Classified as hazardous waste due to their toxic nature, spent catalysts face stringent scrutiny from environmental regulators, compelling refiners to adopt safe and sustainable disposal strategies.^96^ Traditional landfill disposal is now widely deemed unacceptable, as the metals within the catalysts can leach into groundwater, generating environmentally damaging leachate and potentially releasing toxic gases upon contact with water.^97^ Consequently, encapsulation is often required prior to disposal to prevent contaminant release. Alternative management strategies focus on valorization through regeneration, rejuvenation, and reuse either in fresh catalyst formulations or in less severe hydrotreating units before final disposal. For metal recovery, two principal methodologies are employed: pyrometallurgy and hydrometallurgy. Pyrometallurgical processing involves high-temperature treatment to recover valuable metals and dissociate hydrocarbons, a process that is energy-intensive, costly, and generates hazardous flue gases requiring sophisticated cleaning systems. In contrast, hydrometallurgy, or leaching, operates at substantially lower temperatures using aqueous chemicals (acids or bases) to selectively dissolve metals from the waste matrix. Research by Marafi and Stanislaus,^98^ demonstrated that ultrasonic agitation significantly enhances the extraction efficiency of valuable metals (Mo, V, Ni) from spent catalysts during acid leaching. Their comparative study revealed that citric acid, an organic acid, outperformed inorganic H_2_SO_4_ in metal leaching efficacy. The superior performance of citric acid is attributed to a dual mechanism involving liquid–solid reactions for metal detachment and the complexation of dissolved metals with citrate anions, where the stability and solubility of the resulting metal complexes are key determinants. Ultrasonic vibration proved markedly more effective than conventional mechanical stirring, enabling over 95% extraction of Mo, V, and Ni at a moderate 60 °C in a short duration, thereby presenting a more efficient and less energy-intensive route for spent catalyst reclamation.

## 5. Bifunctional mechanism of DRM over Ni-based catalysts

The dry reforming of methane (DRM) over promoted nickel catalysts follows a bifunctional mechanism. Methane is activated on nickel metal sites (modified by Pt, Mo, Fe), while carbon dioxide is activated on the redox-active support (CeO_2_) and on promoter-induced oxygen vacancies. Al_2_O_3_ serves as a high-surface-area structural support that disperses the active phases but does not directly participate in CO_2_ activation.

### 5.1. Methane activation on nickel sites

On the surface of nickel nanoparticles (alloyed with Pt, Mo, Fe), methane adsorbs and dissociates stepwise:^99^10
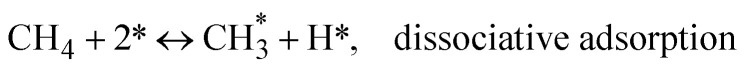
11
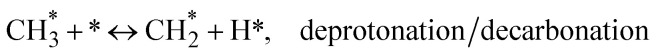
12
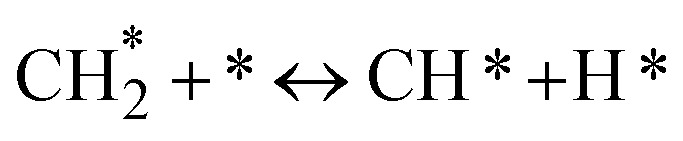

13 CH* + * ↔ C* + H*,

Here, (*) denotes an empty active site on the Ni–MO_*x*_ alloy. The overall dehydrogenation is:

14 CH_4_ + 5* ↔ C* + 4H*,

Hydrogen atoms recombine and desorb as H_2_.

### 5.2. Carbon dioxide adsorption and activation on support and promoters

CO_2_ can adsorb on different sites: on CeO_2_ oxygen vacancies (Oν), on promoter metals, and at the metal–support interface.15
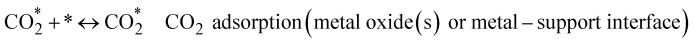

16 CO_2_ + O_ν_ ↔ CO* + O^−2^ (CO_2_ adsorption on oxygen vacancies of CeO_2_)

17
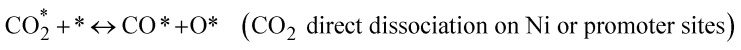
18
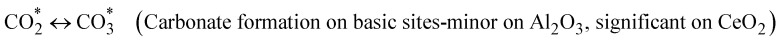
19
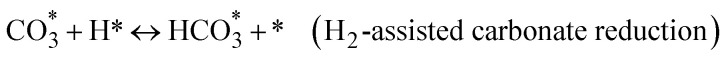
20
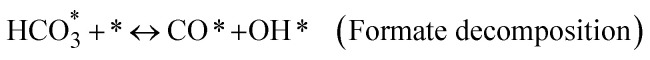
21
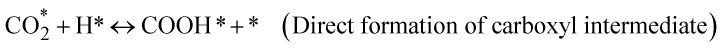

22 COOH* + * ↔ +CO* + H* (carboxyl decomposition)

In the Pt/Mo–Fe/Ni/Al_2_O_3_–CeO_2_ catalyst, the most important pathway is *via* oxygen vacancies (eqn (16)), as this generates mobile lattice oxygen that can migrate to the Ni interface.

### 5.3. Oxidation of CH_*x*_ intermediates and carbon removal

The adsorbed carbon (C) from methane dissociation can be oxidized by oxygen atoms (O) or by lattice oxygen (OLT) migrating from CeO_2_.

23 C* + O* ↔ CO* + * (Ni–MO_*x*_ sites)

24 C* + O_LT_ ↔ CO + O_LT_ (Ni–MO_*x*_ sites)

Similarly, CH_*x*_ species can be oxidized stepwise:25
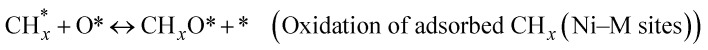

26 CH_*x*_O* + x* ↔ CO* + *x*H* (Ni–MO_*x*_ sites)

These reactions convert carbon into CO, which desorbs, thereby suppressing coke formation.

### 5.4. Desorption and reoxidation steps

The final products leave the surface, and oxygen vacancies are replenished:

27 CO_2_ + O_LT−1_ ↔ O_LT_ + CO (Ni–MO_*x*_ sites) vacancy filling

28
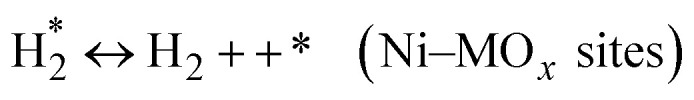

29 CO* ↔ CO + + * (Ni–MO_*x*_ sites)

### 5.5. Mechanistic insights into coke suppression

**Fig. 10:**
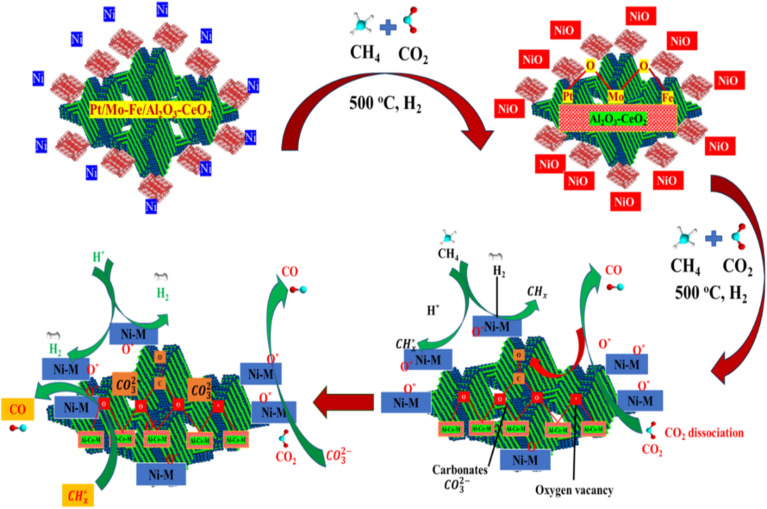
Scheme showing the routes taken during the synthesis, reduction, and reaction mechanism of the catalyst for DRM over Pt/Mo–Fe/Ni/Al_2_O_3_–CeO_2_.

Fan *et al.*^76^ postulated a different reaction mechanism for catalysts with basic supports, such as CeO_2_, in which CO_2_ activation occurs on the surface of the support rather than the metal active site. In this mechanism, CO_2_ is adsorbed vicinity the metal particles on the catalyst support, CO_2_ (g) = CO_2_ (support), resulting in the carbonate species CO_2_ (support) + O^2−^ = CO_3_^2−^ (support). The hydrogen was then used to reduce the carbonate (CO_3_^2−^ (support) + 2H = HCO_2_^−^ (support) + OH^−^) to produce CO.^100^ According to the theory, CO_2_ is adsorbing onto promoters like Ce or CeO and dissociate into CO and O (eqn (16)). Following this, carbon from the decompose of CH_4_ is deposited on the catalyst's active sites where it reacts with the adsorbed oxygen on the promoter to generate CO (O* + C* → CO*). Fig. 10 presents a schematic illustration of the proposed reaction mechanism for syngas production *via* dry reforming of methane (DRM) over the Pt/Mo–Fe/Ni/Al_2_O_3_–CeO_2_ catalyst. The diagram integrates three stages: catalyst synthesis, reduction pretreatment, and the actual DRM reaction pathway. As shown in the Fig. 10, the DRM process begins with the adsorption of the reactant gases, CH_4_ and CO_2_, onto the catalyst surface. CH_4_ adsorbs on the Ni–MO_*x*_ interface (where M = Mo, Fe, and Pt), where it undergoes stepwise dehydrogenation to produce hydrogen atoms and CH_*x*_ species. These CH_*x*_ intermediates remain bound to the nickel sites. Simultaneously, CO_2_ adsorbs on oxygen vacancies present on the CeO_2_ support and on the MoO_*x*_/FeO_*x*_ promoter phases. At these vacancy sites, CO_2_ dissociates into CO and an oxygen atom (O). The resulting oxygen species are highly mobile and can migrate to the Ni-support interface. The key anti-coking step occurs when the adsorbed carbon (C*) from CH_4_ decomposition reacts with the mobile oxygen (OLT) originating from CeO_2_ or the promoters. This reaction produces CO, which desorbs from the catalyst surface, effectively removing the carbon precursor before it can form graphitic whiskers or encapsulating films. The oxygen vacancies consumed during this process are continuously replenished by CO_2_, maintaining the redox cycle of the support. The role of Al_2_O_3_ in this catalyst is primarily structural; it provides a high surface area for dispersing the active Ni and promoter phases but does not directly participate in CO_2_ activation. The CeO_2_ component is critical for generating and maintaining oxygen vacancies, while the Pt, Mo, and Fe promoters enhance the reducibility of Ni, increase the concentration of oxygen vacancies, and promote hydrogen spillover. This cooperative bifunctional mechanism explains the superior coking resistance and long-term stability observed for the Pt/Mo–Fe/Ni/Al_2_O_3_–CeO_2_ catalyst compared to unpromoted Ni/Al_2_O_3_–CeO_2_ systems. Detailed experimental evidence supporting this scheme, including the temperature-programmed reduction of H_2_ (H_2_-TPR), X-ray photoelectron spectroscopy (XPS), and the temperature-programmed desorption of CO_2_ (CO_2_-TPD) data, has been reported previously.^80,101,102^

## 6. Redox-type metal oxide catalysts

**Fig. 11:**
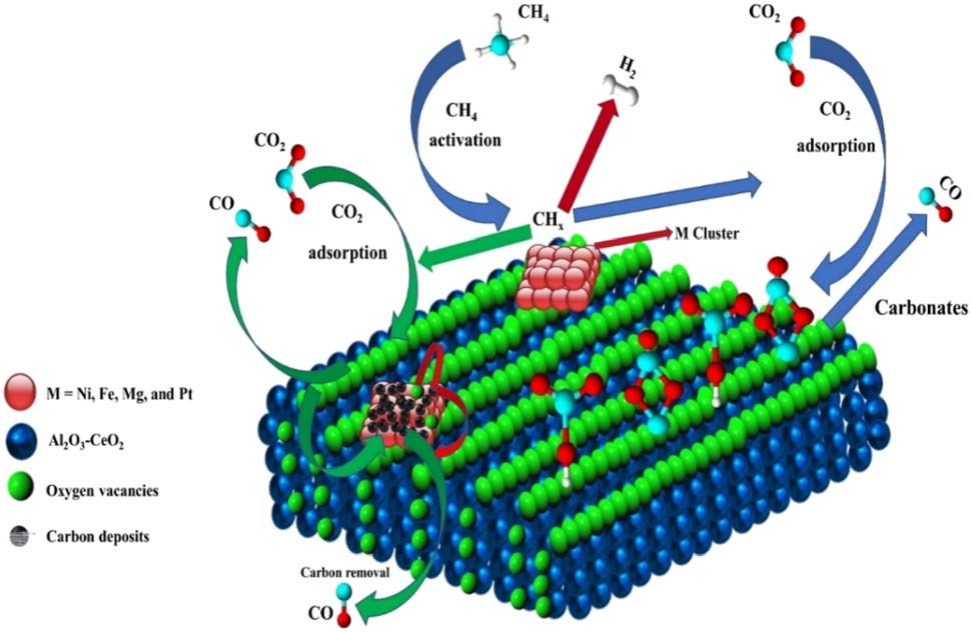
Schematic depiction of the bifunctional DRM reaction mechanism over a Ni-based catalyst. CH_4_ is activated on Ni metal sites (producing H and CH_*x*_ species), while CO_2_ is activated on the support or promoter (producing CO and O). Oxygen species migrate to the Ni–support interface to gasify carbon precursors, suppressing coke formation.

**Fig. 12:**
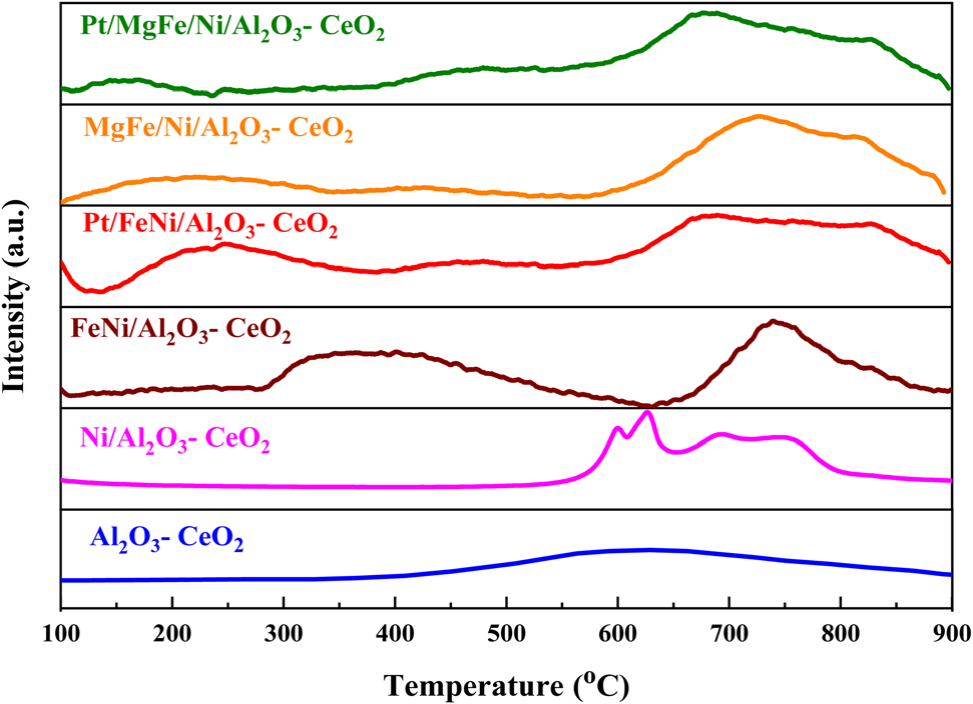
H_2_-TPR profiles of Ni-based Al_2_O_3_–CeO_2_ composite catalysts. Bi-and trimetallic samples (*e.g.*, Pt/Mg–Fe/Ni) show superior reducibility (lower reduction temperatures) compared to monometallic Ni, indicating enhanced metal–support interaction and improved coke resistance.^99^

**Fig. 13:**
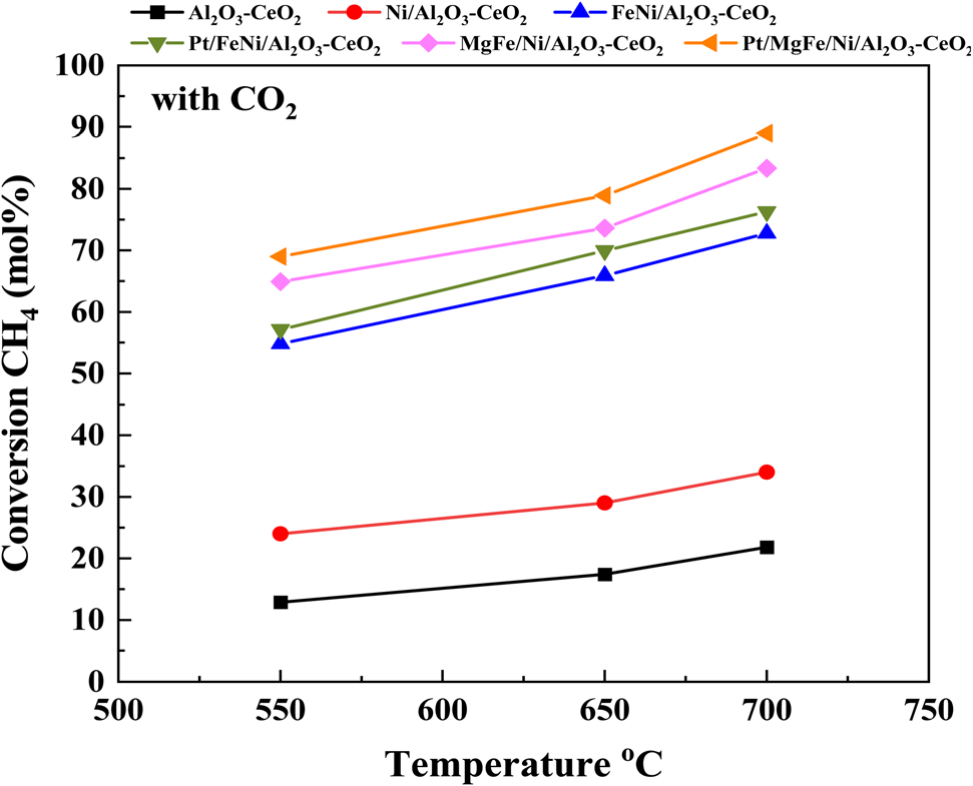
CH_4_ and CO_2_ conversion *vs.* reaction temperature. Reaction condition; reaction temperature 550–700 °C; *P* = 1 bar; feed gas CH_4_/CO_2_ = 50/50 (with CO_2_), flow rate = 60 mL min^−1^, WHSV = 12 000 mL g_cat_^−1^ h^−1^.^99^

The strategic incorporation of metal oxide promoters is fundamental to enhancing the performance of catalysts in the dry reforming of methane (DRM). These promoters significantly improve catalytic efficacy by augmenting surface basicity, generating oxygen vacancies, enhancing redox properties, and improving nickel particle dispersion. The reaction mechanism, as depicted in Fig. 11, involves bifunctional activation where CH_4_ is activated on Ni sites while CO_2_ adsorption and activation occur on the catalyst surface. Enhancing catalyst basicity accelerates CO_2_ activation, thereby substantially influencing overall performance. Previous studies.^99^ have confirmed that metal oxide incorporation effectively increases surface basicity and CO_2_ adsorption capacity, as demonstrated in Pt/MgFe/Ni/CeO_2_–Al_2_O_3_ catalysts. H_2_-TPR analysis further suggests that hydrogen spillover effects facilitate the reduction of metal oxides due to unique interactions between Ni and promoter species, as illustrated in Fig. 12 where bi/tri-metallic samples show superior reducibility compared to monometallic counterparts. The Al_2_O_3_–CeO_2_ composite support is crucial for optimizing active site dispersion and enhancing catalytic stability. Temperature-programmed desorption of CO_2_ (CO_2_-TPD) reveals that adsorbed CO_2_ forms both bidentate and monodentate carbonate species, with bidentate carbonates being more effective for converting CH_*x*_ intermediates and facilitating carbon deposit removal. The presence of oxygen vacancies and robust redox properties is critical for superior performance. Metal oxide-modified catalysts exhibit enhanced redox characteristics and higher oxygen vacancy density, supplying active oxygen species for reactant activation and carbon removal. The uniform dispersion of Ni species, aided by the confinement effect of metal oxides, positively influences anti-coking behavior by hindering carbon nucleation. As shown in Fig. 13, the optimal DRM activity at 700 °C for Pt/Mg–Fe/Ni-based Al_2_O_3_–CeO_2_ catalysts is attributed to enhanced methane decomposition coupled with efficient oxidation of intermediate carbon by activated CO_2_.^99,103^

## 7 Studying of CO_2_ and CH_4_ activation using DFT calculation

**Fig. 14:**
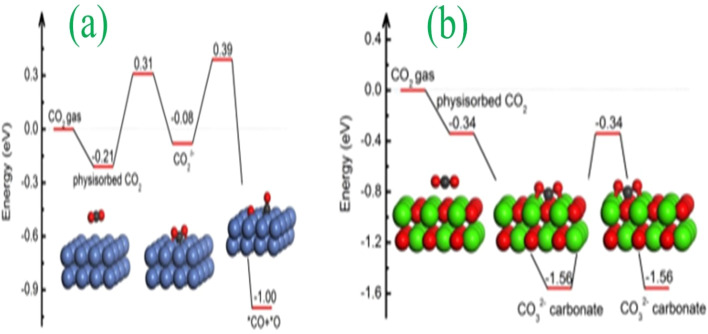
Depicts the minimal reaction pathways for CO_2_ adsorption (containing the physisorbed CO_2_ and CO_2_^*δ*−^ state) and dissociation on (a) Ni(111) surfaces and CO_2_ adsorption (including the physisorbed CO_2_ and CO_2_^2−^ carbonate (R0)) and diffusion on (b) CaO(100) surfaces. The blue, red, green, and black balls represent Ni, O, Ca, and C atoms, respectively.^111^

**Fig. 15:**
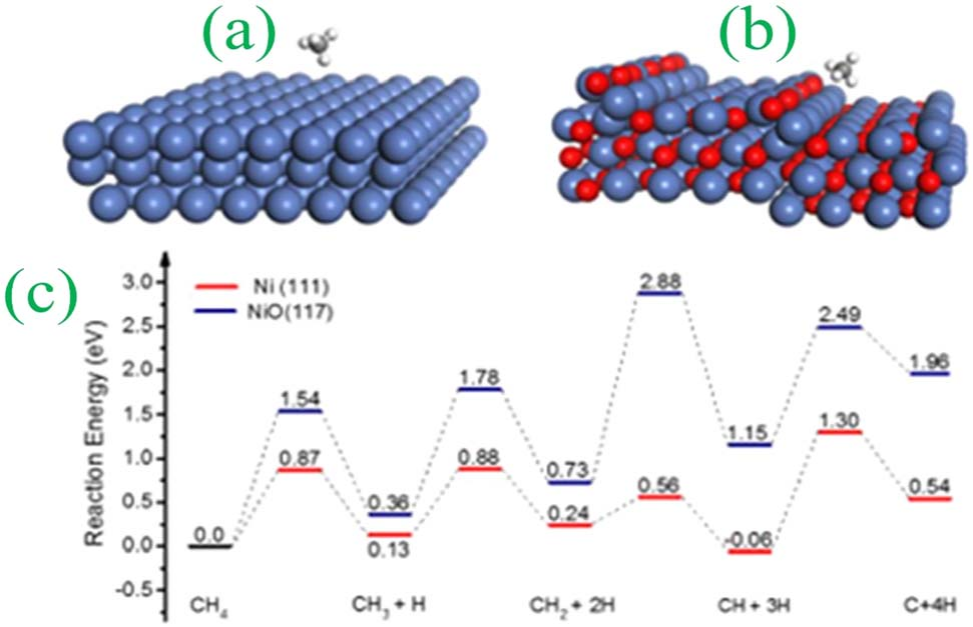
The initial state of (a) Ni(111) and (b) NiO adsorption of CH_4_ (117). (c) The least energy paths for CH_4_ dehydrogenation on Ni(111) and NiO(117).^111^

This section summarizes key computational studies from the literature that provide atomistic insights into DRM mechanisms, with an emphasis on Ni-based systems and coke formation. Shaikhutdinov *et al.*,^104^ explored the adsorption and activation of CH_4_ and CO_2_ on Ni(111), CaO(100), and a CaO step site. On the Ni(111) surface, CH_4_ is physisorbed on the top Ni site with an adsorption energy of −0.26 eV. The Ni(111) surface facilitates the dehydrogenation of CH_4_, with an activation energy (*E*_act_) for the first dehydrogenation step of 0.87 eV, which aligns with prior studies.^105^ For CO_2_ adsorption on Ni(111), two distinct modes are observed: physisorbed CO_2_ and CO_2_^*δ*−^ states, as depicted in Fig. 14a. The adsorption energy for physisorbed CO_2_ is approximately −0.21 eV, with a bond length (LC–O) of 1.18 Å and a bond angle (*θ*_O_–C–O) of 179.7°, similar to the CO_2_ gas state (LC–O = 1.16 Å, *θ*_O_–C–O = 180°). In contrast, the CO_2_^*δ*−^ state, which is adsorbed on the top site of Ni(111) and forms a Ni–C bond, shows a bent CO_2_ with an LC–O of 1.28 Å and a *θ*_O_–C–O of 131.8°, consistent with previous theoretical studies.^106^ The bending of CO_2_ increases the adsorption energy of the CO_2_^*δ*−^ state by 0.13 eV compared to the physisorbed CO_2_, indicating that the transition from physisorbed to CO_2_^*δ*−^ state is endothermic with a reaction energy (Δ*E*) of 0.13 eV. As illustrated in Fig. 14a, the activation energy required for the transition from physisorbed CO_2_ to the CO_2_^*δ*−^ state is calculated to be 0.52 eV. The CO_2_^*δ*−^ state readily dissociates into CO and O species, which occupy the hollow sites on Ni(111), with an activation energy of 0.47 eV (Fig. 14a), in close agreement with a previously reported value of 0.43 eV.^106^ This indicates that Ni nanoparticles are effective in both CH_4_ dehydrogenation and CO_2_ dissociation, consistent with earlier theoretical and experimental findings.^107,108^ For CH_4_ on CaO(100), the physisorption adsorption energy is approximately −0.20 eV. CO_2_ adsorption on CaO(100) reveals two modes: physisorbed CO_2_ and CO_2_^*δ*−^ carbonate, as shown in Fig. 14b. The physisorbed CO_2_, with a bond length (LC–O) of 1.18 Å and a bond angle (*θ*_O_–C–O) of 176°, exhibits a geometry similar to that of CO_2_ in the gas phase, and has an adsorption energy of −0.34 eV. For the CO_3_^2−^ carbonate on CaO(100), two distinct configurations are identified:^109^ one (R0) is where the oxygen atom of CO_3_^2−^ carbonate points towards to oxygen atom of support, while the other (R45) is that CO_3_^2−^ carbonate in the R0 state is rotated about 45° to align with the Ca atom of support. Their adsorption energies are −1.56 eV and −1.59 eV respectively, −1.48 eV and −1.51 eV lower than that of the CO_2_^*δ*−^ state on Ni(111). Besides the CaO(100), Shaikhutdinov *et al.*, also considered the influence of CaO step on CH_4_ and CO_2_ adsorption (Fig. 15). On the CaO step, CH_4_ physisorbs with an adsorption energy of −0.22 eV, whereas CO_2_ is strongly bound with an adsorption energy of −2.78 eV, consistent with previous findings.^110^ Therefore, CO_2_ thermodynamically prefers to adsorb on CaO rather than on Ni surfaces. When examining CH_4_ dehydrogenation and CO_2_ dissociation on CaO(100), it was found that CH_4_ dehydrogenation is highly endothermic, with a reaction energy (Δ*E*) of 3.95 eV, making it difficult to occur on CaO(100). Similarly, CO_2_ activation on CaO(100) is challenging due to the weak interaction between CO_2_ and CaO(100) (*E*_abs_ = −0.33 eV), with a reaction energy of 4.35 eV. However, CO_2_ exhibits moderate mobility on CaO(100). As shown in Fig. 14b, he diffusion energy barrier for the R0 CO_3_^2−^ carbonate on CaO(100) is calculated to be 1.22 eV, which is 0.34 eV lower than the desorption energy barrier (1.56 eV). Based on these DFT calculations, it can be concluded that the CaO promoter does not facilitate CH_4_ hydrogenation or CO_2_ dissociation and primarily functions as a CO_2_ sorbent. Due to the strong interaction between CO_2_ and the CaO surface, it is also inferred that large amounts of CaO serve effectively in this role.

## 8. Kinetic assessment of the DRM reaction

**Fig. 16:**
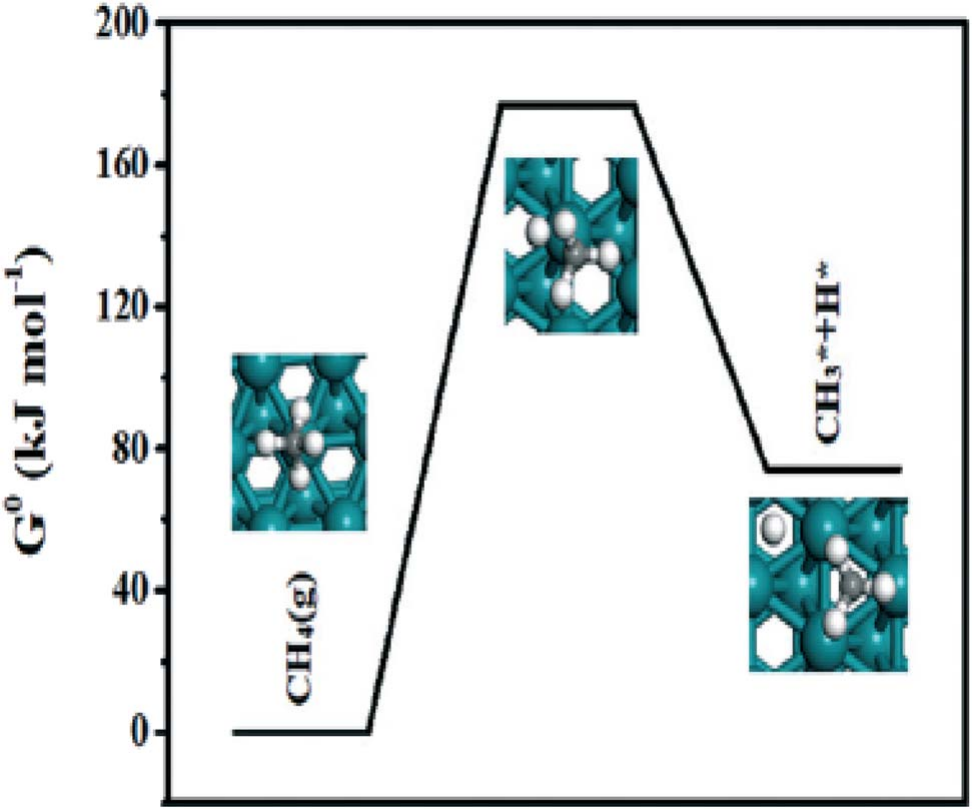
Free energy profile of CH_4_ dissociation on the Ru(0001) surface at 973.15 K.^112^

**Fig. 17:**
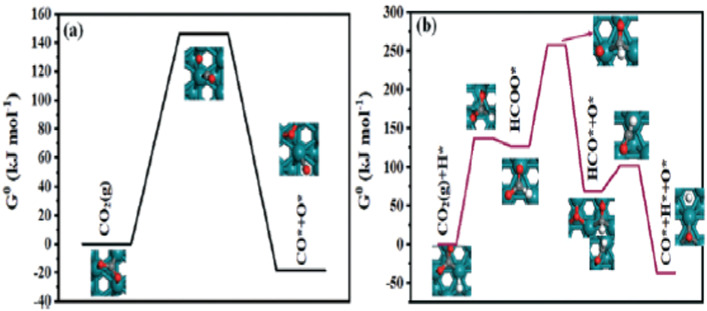
Free energy profiles of CO_2_ activation on the Ru(0001) surface at 973.15 K. Two pathways are possible for C–O activation: direct (a) and hydrogen-assisted *via* HCOO* intermediate (b).^112^

**Fig. 18:**
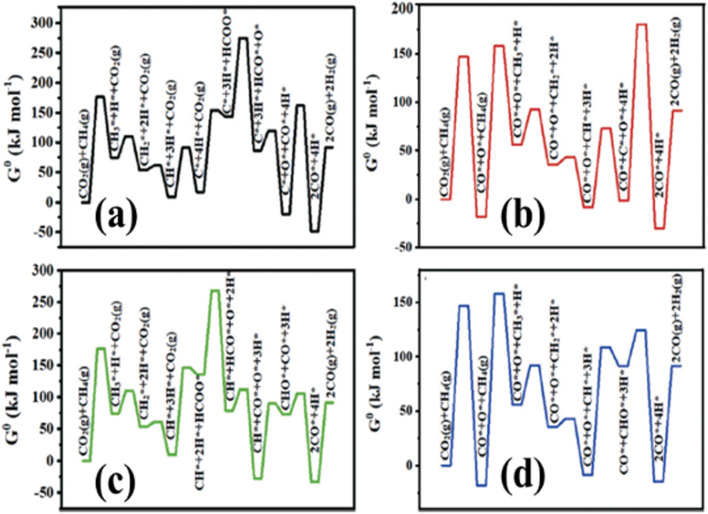
Free energy profiles of the dry reforming of methane on the Ru(0001) surface at 973.15 K. Four pathways are possible for dry reforming: (a) hydrogen-assisted CO_2_ dissociation with C–O oxidation, (b) CO_2_ direct dissociation with C–O oxidation, (c) hydrogen-assisted CO_2_ dissociation with CH–O oxidation, and (d) CO_2_ direct dissociation with CH–O oxidation.^112^

**Fig. 19:**
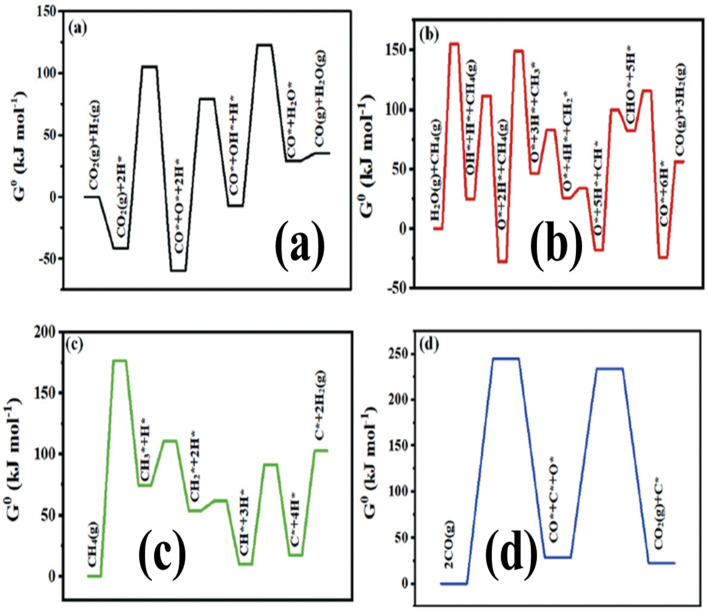
Free energy profiles of the competitive reaction of DRM on the Ru(0001) surface at 973.15 K. Four pathways are possible for the competitive reaction: RWGS (a), SRM (b), methane cracking (c), and the Boudouard reaction (d) of DRM.^112^

Wang *et al.*,^112^ investigated two key processes. Fig. 16 illustrates the activation energy for CH_4_. The activation free energy for CH_4_ activation was determined to be 176 kJ mol^−1^, and the standard free energy of the CH_4_ activation reaction at 973.15 K (a typical DRM reaction temperature^113^) Δ*rG*_0_) was 74 kJ mol^−1^. For the activation of CO_2_, two potential pathways were considered: (1) direct CO_2_ dissociation, and (2) hydrogen-assisted CO_2_ dissociation. The mechanism for hydrogen-assisted CO_2_ dissociation is as follows: 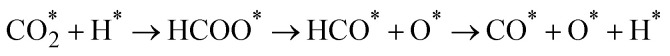. Numerous studies have indicated that an alternative mechanism for hydrogen-assisted CO_2_ dissociation 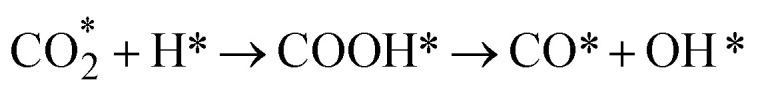) is not the favorable pathway for CO_2_ activation on the Ru(0001) surface,^114^ and thus it was excluded from this study to simplify the calculations. Fig. 17 illustrates two different pathways for CO_2_ activation. The free energy spans for direct CO_2_ dissociation (Fig. 17a) and hydrogen-assisted CO_2_ dissociation (Fig. 17b) were 147 and 258 kJ mol^−1^, respectively. Evidently, direct dissociation exhibited a lower activation energy, making it the primary mechanism for CO_2_ dissociation on Ru(0001). In previous studies, CO_2_ activation on Ni catalysts was also examined through these two pathways. For Ni catalysts, there are two possible routes for hydrogen-assisted CO_2_ dissociation: (1) *via* the COOH* intermediate, and (2) *via* the HCOO* intermediate.^115^ However, similar to Ru catalysts, the direct dissociation of CO_2_ is the predominant pathway for CO_2_ dissociation on Ni surfaces,^105^ which is the same as on Ru catalysts. Moreover, on the surfaces of Ru and Ni catalysts, the activation energy required for CO_2_ dissociation is lower than that for CH_4_ dissociation. The difference in energy barriers for CH_4_ and CO_2_ dissociation is more pronounced on Ru compared to Ni (30 kJ mol^−1^*vs.* 20 kJ mol^−1^, it might be expected that Ru would strongly resist carbon deposition. In the previous work showed that the pre-adsorbed oxygen atom inhibited the C–H bond dissociation process on the Ru surface,^116^ so the 
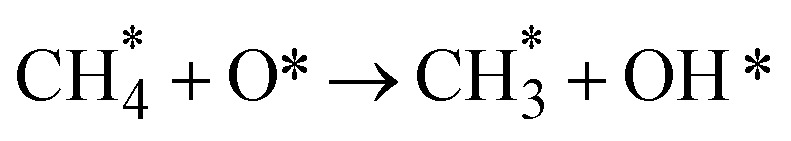
 step was not considered in the present work. Through the previous analysis, direct dissociation is undoubtedly the optimal path for CO_2_ dissociation. Also, the O* produced by the direct dissociation of CO_2_ is naturally considered to be the main oxidant. OH* is also often considered the main oxidant, but Hensen *et al.*, mainly considered O* because the previous research provided us with evidence that O* and OH* have a similar oxidizing capacity or O* has a more powerful oxidation ability.^117^ Regarding the formation of the C–O bond, the free energy barriers for C–O oxidation and CH–O oxidation were found to be 181 and 100 kJ mol^−1^, respectively. This suggests that CH–O oxidation might be the primary route for C–O bond formation. However, considering that CH–O oxidation has a higher free energy barrier than CH* dissociation, both pathways for C–O bond formation should be taken into account. Additionally, it is important to note that the Boudouard reaction (
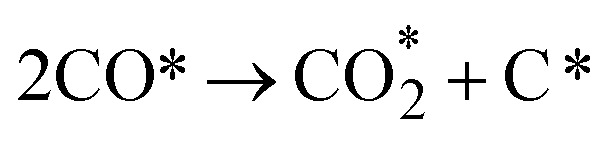
) occurs in two steps rather than one: first, 2CO* → O* + C* + CO*, followed by 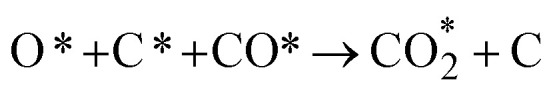. The activation energies for these steps are 270 and 206 kJ mol^−1^, respectively. Based on the activation energies of these elementary reactions, Wang *et al.*, constructed the free energy profiles for the DRM reaction as it proceeds through various pathways (Fig. 18). Illustrates several possible reaction pathways for DRM. At 973.15 K, the DRM reaction is endothermic with a standard free energy of 91 kJ mol^−1^. Based on different CO_2_ dissociation and C–O bond formation mechanisms they identified four distinct pathways: (a) hydrogen-assisted CO_2_ dissociation with C–O oxidation (Fig. 18a), direct CO_2_ dissociation with C–O oxidation (Fig. 18b), hydrogen-assisted CO_2_ dissociation with CH–O oxidation pathway (Fig. 18c), direct CO_2_ dissociation with CH–O oxidation (Fig. 18d). The overall free energy spans for these paths were 275, 181, 267, and 158 kJ mol^−1^, respectively. Clearly, pathway (d), direct CO_2_ dissociation with CH–O oxidation, emerged as the most favorable for the DRM reaction on the Ru(0001) surface at 973.15 K. This finding corroborates our earlier conclusions regarding the preferred CO_2_ dissociation and C–O bond formation mechanisms. The DFT results assume sequential reaction steps, so Fig. 18 focuses on the scenario where the direct dissociation of CO_2_ precedes CH_4_ activation. The free energy profiles of the competitive reactions in DRM are illustrated in Fig. 19. These profiles consider only favorable pathways, namely direct CO_2_ dissociation and CH–O oxidation. The free energy spans for the reverse water–gas shift (RWGS) reaction Fig. 19, steam reforming of methane (SRM) (Fig. 19b), CH_4_ cracking (Fig. 19a), SRM (Fig. 19b), CH_4_ cracking (Fig. 19c), and the Boudouard reaction (Fig. 19d) were 123, 155, 176, and 245 kJ mol^−1^, respectively. For the Boudouard reaction, its direct pathway can contribute to C* formation, while previous studies.^118^ Through this approach, the energy barrier for CO_2_ activation was 223 kJ mol^−1^, lower than that for hydrogen-assisted CO_2_ dissociation. The order of the free energy spans for the DRM reaction and its competitive reactions on the Ru(0001) surface was as follows: RWGS reaction < SRM reaction ≈ DRM reaction < C* formation reaction (CH cracking and the Boudouard reaction). This sequence effectively explains why the RWGS reaction always accompanies the DRM reaction on the Ru catalyst surface and why the Ru catalyst is resistant to carbon deposition. On the Ni(111) surface, the order was: C* formation reaction ≈ RWGS reaction < SRM reaction ≈ DRM reaction.^118^ This significant difference in the order of free energy spans between the Ru and Ni surfaces is primarily related to the C* formation reaction. For the Ru catalyst, the C* formation reaction was not dominant, whereas for the Ni catalyst, it had the lowest free energy span among all reactions. Consequently, the Ru catalyst exhibited a stronger ability to prevent coke formation compared to the Ni catalyst.

## 9. Industrial perspective: scalability, catalyst cost, and long-term stability

### 9.1. Catalyst cost

The Table 4 showing estimated catalyst costs (USD/kg, assuming 10 wt% Ni on Al_2_O_3_ with 1 wt% promoter where applicable). The cost estimates are derived from:

**Table 4:** Estimated catalyst cost for different formulations

Catalyst	Estimated cost (USD per kg)	Ref.
Ni/Al_2_O_3_	30–50	4
Ni–Co or Ni–Fe	50–80	51 and 67
Ni–Mo	80–120	80
Ni–Ru	500–800	62
Ni–Pt	600–1000	57, 119 and 120

#### 9.1.1 General Ni-based catalyst cost

Ni-only catalysts are widely recognized as the most cost-effective option. Several review sources emphasize that the low price of nickel compared to noble metals is the primary reason Ni remains the preferred active metal for DRM, despite its tendency to coke. The global market price for nickel reforming catalysts is reported to be around US$ 85 per kg.

#### 9.1.2 Noble metal promoter costs

The high cost of noble metals (Pt, Ru) is a major barrier to their industrial application. Even a small amount of Pt or Ru increases the catalyst cost by an order of magnitude.

Summery, noble metal promoters (Pt, Ru) increase catalyst cost by 10–20 times compared to Ni-only catalysts. For a large-scale DRM plant, this difference amounts to millions of dollars. Therefore, non-noble promoted catalysts (Fe, Co, Mo) are the only economically viable option for industrial application.^67,73^

### 9.2. Industrial scalability challenges

Three synthesis methods and their scalability were listed in Table 5.

**Table 5:** Comparison of synthesis methods for Ni-based DRM catalysts: scalability and limitations

Method	Scalability	Key limitation	Ref.
Impregnation	High	Broad Ni particle size distribution, weaker MSI	58, 64 and 69
Co-precipitation	Moderate	PH-sensitive, batch -to-batch variability	50 and 121
Sol–gel/hydrothermal	Low	High solvent volume, long aging time, waste disposal	23

### 9.3. Long-term stability

The explicitly state the gap between laboratory research and industrial requirement were displayed in Table 6.

**Table 6:** Quantitative comparison of carbon deposition rates, stability duration, and carbon type for selected Ni-based DRM catalysts

Catalyst	Coke rate mg C g_cat_^−1^ h^−1^	Stability (h)	Carbon type	Ref.
Ni/γ-Al_2_O_3_	15.4	20	Whisker + encapsulating	51
Ni/La_2_O_3_–ZrO_2_	3.8	100	Amorphous	44
Pt–Ni/Mg/(Ce, Zr)O_2_	<0.5	100	Trace nanotubes	58
Ni/γ-Al_2_O_3_ (steam pretreated)	3.6	200	Amorphous	94
Ni/CeO_2_	6	50	Mostly amorphous	48
Ni/MgO(Ni_0.1_Mg_0.9_O)	8.2	24	Encapsulating	50
Industrial target	<0.1	>8000	No graphitic carbon	—

#### 9.3.1 Industrial target

>8000 h on stream (≈1 year) with coke deposition rate < 0.1 mg C g_cat_^−1^ h^−1^.

#### 9.3.2 Best laboratory catalysts (Table 6)

100–500 h at 1 bar, with coke rates between 0.5 and 15 mg C g_cat_^−1^ h^−1^.

## 10. Conclusion

This comprehensive review has systematically charted the progress and persistent challenges in DRM over Ni-based catalysts. The following key insights and gaps have been identified.

• Mechanistic consensus: redox-active supports (CeO_2_, ZrO_2_) outperform basic supports (MgO, La_2_O_3_) for long-term stability due to mobile lattice oxygen that gasifies carbon. Basic supports excel in CO_2_ conversion at lower temperatures *via* carbonate intermediates.

• Quantitative benchmarks: coke deposition rates range from <0.5 to >15 mg C g_cat^−1^ h^−1^, and catalyst stability from 20 to 200 h at 1 bar – two orders of magnitude below industrial targets (>8000 h, <0.1 mg C g_cat^−1^ h^−1^).

• Contradictions remain open: the dominant coke source (CH_4_ cracking *vs.* Boudouard) and optimal promoter (Pt *vs.* Ru) depend on Ni particle size, support basicity, and reaction conditions. No universal descriptor exists.

• Industrial barriers: noble metal promoters increase catalyst cost significantly. Scalable synthesis methods produce lower performance catalysts. High pressure coking and H_2_S poisoning are understudied.

• Future directions: high throughput screening, machine learning, pilot-scale tests (>10 bar, H_2_S, >1000 h), low cost non-noble catalysts, and robust regeneration protocols are urgently needed.

## Conflicts of interest

The authors declare no competing financial interest.

## Data Availability

All data generated or analyzed during this study are included in this published article in the main manuscript.
